# Super-stable cyanine@albumin fluorophore for enhanced NIR-II bioimaging

**DOI:** 10.7150/thno.71443

**Published:** 2022-05-26

**Authors:** Lang Bai, Zhubin Hu, Tianyang Han, Yajun Wang, Jiajun Xu, Guanyu Jiang, Xin Feng, Bin Sun, Xiangping Liu, Rui Tian, Haitao Sun, Songling Zhang, Xiaoyuan Chen, Shoujun Zhu

**Affiliations:** 1 State Key Laboratory of Supramolecular Structure and Materials, College of Chemistry, Jilin University, Changchun 130012, P.R. China; 2 Joint Laboratory of Opto-Functional Theranostics in Medicine and Chemistry, The First Hospital of Jilin University, Changchun, 130021, P.R. China; 3 State Key Laboratory of Precision Spectroscopy, School of Physics and Electronic Science, East China Normal University, Shanghai 200062, P.R. China; 4 State Key Laboratory of Molecular Vaccinology and Molecular Diagnostics & Center for Molecular Imaging and Translational Medicine School of Public Health, Xiamen University, Xiamen, 361102, P.R. China; 5 Collaborative Innovation Center of Extreme Optics, Shanxi University, Taiyuan, Shanxi 030006, China; 6 Department of Obstetrics and Gynecology, The First Hospital of Jilin University, Changchun, 130021, P.R. China.; 7 Yong Loo Lin School of Medicine and Faculty of Engineering, National University of Singapore, 117597, Singapore, Singapore

**Keywords:** NIR-II imaging, cyanine dye, albumin, super-stable NIR-II probe, covalent bond

## Abstract

Near-infrared-II (NIR-II) dyes could be encapsulated by either exogenous or endogenous albumin to form stable complexes for deep tissue bioimaging. However, we still lack a complete understanding of the interaction mechanism of the dye@albumin complex. Studying this principle is essential to guide efficient dye synthesis and develop NIR-II probes with improved brightness, photostability, *etc*.

**Methods:** Here, we screen and test the optical and chemical properties of dye@albumin fluorophores, and systematically investigate the binding sites and the relationship between dye structures and binding degree. Super-stable cyanine dye@albumin fluorophores are rationally obtained, and we also evaluate their pharmacokinetics and long-lasting NIR-II imaging abilities.

**Results:** We identify several key parameters of cyanine dyes governing the supramolecular/covalent binding to albumin, including a six-membered ring with chlorine (Cl), the small size of side groups, and relatively high hydrophobicity. The tailored fluorophore (IR-780@albumin) exhibits much-improved photostability, serving as a long-lasting imaging probe for NIR-II bioimaging.

**Conclusion:** Our study reveals that the chloride-containing cyanine dyes with the above-screened chemical structure (e.g. IR-780) could be lodged into albumin more efficiently, producing a much more stable fluorescent probe. Our finding partly solves the photobleaching issue of clinically-available cyanine dyes, enriching the probe library for NIR-II bioimaging and imaging-guided surgery.

## 1. Introduction

The near-infrared-II (NIR-II) bioimaging affords high-quality imaging capability attributing to light attenuation and scattering as well as background autofluorescence, which all decrease as the wavelength increases 1-9. However, current NIR-II probes still suffer from drawbacks in terms of relatively low brightness and narrow imaging window. The interaction between dyes and proteins in the physiological environment also delivers uncontrolled pharmacokinetics and long-term biotoxicity after intravenous administration of NIR-II fluorophores 10, 11. The controllable pharmacokinetic is essential for both desired imaging time window and eliminating exogenous biotoxicity concerns 12, 13. When the NIR-II probes enter the blood circulation, they will be governed by specific biomolecules and biological processes, finally enzymatically degraded or eliminated from the body 14-17. The probe-biology interaction depends on the molecular structure of the administered probes, including size, polarity, functional groups, hydrophilicity/hydrophobicity, *etc* 17. Thus, it is still challenging to clarify the interaction mechanism between NIR dye and protein (*e.g.* albumin), and predict the brightness enhancement and pharmacokinetics of the dye-protein complex.

The exogenous albumin can serve as a versatile carrier for prolonging the *in vivo* circulation time of small molecules, producing stable and long-lasting drug and/or imaging probes 18-23. The interaction between small molecules and albumin essentially may include both covalent conjugation and supramolecular encapsulation. The interaction could produce a dynamically stable probe, effectively protecting the dye molecules from endogenous enzymolysis and lengthening the imaging window 24. Notably, the brightness or quantum yield (QY) of the dye-protein probe is remarkedly improved by the confinement of the dye in the hydrophobic pocket of albumin 24-30. The dye@albumin probe prominently avoids the intramolecular aggregation of the molecular dyes, thus reducing the self-quenching issue 24, 27. Meanwhile, albumin chaperoned dye also yields satisfactory solubility and biocompatibility, especially for the hydrophobic structures 26.

**Figure 1:**
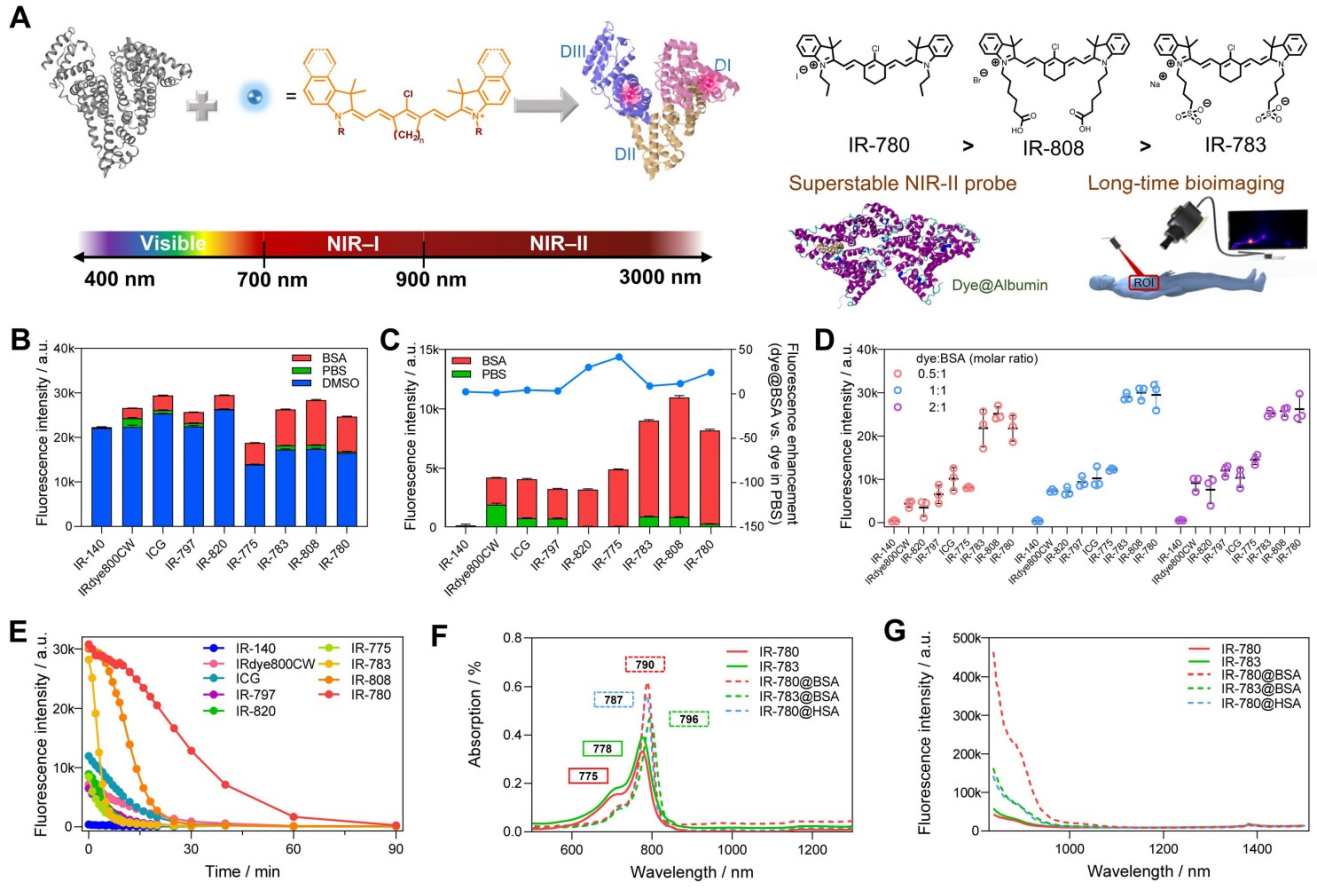
**Screening of dye@albumin fluorophores yielded super-bright IR-780@BSA fluorophore with enhanced photostability.** (A) Simplified schematic for the formation of the dye@albumin fluorophores. (B) Screening of a set of nine dyes with different emission filters and solutions (using the 900+1000 LP detection of NIR-II fluorescence intensity). (C) Selected fluorescence brightness from (B) and brightness enhanced folds of dye@BSA versus those in PBS. (D) Fluorescence brightness of dye@BSA fluorophores at 0.5:1, 1:1, and 2:1 reaction molar ratios. (E) Photostability of dye@BSA fluorophores (1:1 reaction ratio) in NIR-II window under continuous laser irradiation. (F) Absorption spectra and (G) NIR-II emission spectra (808 nm laser excitation) of free dyes and dye@albumin fluorophores. All fluorescence intensity profiles for b-e were performed with 900+1000 nm long-pass filters under 808 nm laser irradiation (70~75 mW/cm^2^). The concentration of tested samples was 10 μM.

Compared with organic synthesis and modification of cyanine dyes 29, 31, 32, albumin interaction affords an alternative strategy to improve their imaging properties, especially for *in vivo* settings 21, 22, 24-30. However, much less is known on the interaction mechanism between cyanine dyes and albumin, and we still lack complete guidance to manufacture a super-stable complex probe with precise control of the combination parameters. Here, we identified the interaction mechanism of cyanine dye and albumin and tailored a super-stable cyanine dye@albumin fluorophore (IR-780@bovine serum albumin, IR-780@BSA) as a long-lasting imaging probe (Figure 1A). The probe demonstrated a comparable brightness to the previously focused IR-783@BSA 24, but with a 10-fold longer imaging window under continuous laser irradiation. Systematic experiments with several settings of cyanine dyes revealed that the supramolecular interaction and covalent binding were two essential steps for the dye@albumin fluorophore. The chloride (Cl)-containing cyanine dye with the favorable chemical structure (*e.g.* IR-780 *vs*. IR-783) could produce a much more stable probe with albumin. The screened super-stable fluorophore provided much improved NIR-II imaging ability by rationally tuning the albumin/albumin fragments and dye structures. Our strategy partly solves the photobleaching issue of clinically available cyanine dyes and affords the comprehensive principle to guide the dye/protein binding and protein-chaperoned probes.

## 2. Results

### 2.1. Rationally screening dye@albumin fluorophores yielded super-bright IR-780@BSA fluorophore with enhanced photostability

Previous studies (including our recent publication) have proved the covalent bonding between chlorine-containing cyanine dyes and albumin 33-36. However, a detailed investigation of the binding mechanism is still lacking to guide the formation of the stable probe. We first screened a library of cyanine dyes to bind with albumin and tested the brightness enhancement under the 900+1000 nm sub-NIR-II window 27, 37, 38. As shown in Figure 1B, by comparing the brightness of 9 dyes diluted in DMSO, PBS, and BSA, we found that cyanine dye@BSA could recover part of brightness compared with them in DMSO, yet cyanine dyes were quenched in PBS due to aggregation caused quench (ACQ) effect 9, 39. The solubility was an important factor to decide their photophysical properties, thus we tested the conservative solubility of dyes and dye@BSA in Table S1. Adding BSA could dramatically increase the solubility of some hydrophobic dyes by binding with BSA. Specifically, the detailed reaction optimization for IR-780 fluorophore as an example was listed in Figure S1, which included incubation temperature, reaction time, and concentration. The optimal reaction condition was as the following: the reaction temperature was 50-60 ºC, the reaction time was > 2 h; the reaction concentration was below 10 μM. It should be noted that cyanine dyes were reported to form non-fluorescent adducts spontaneously over a concentration of 5 µM 40. Thus, we compared different molar concentrations (5 and 10 μM) of 9 dyes, and the results indicated that interacting with BSA could neutralize part of fluorescence quenching (Figure S2). In addition, from the concentration-brightness dependent data (Figure S1B), the brightness values for both 5 and 10 μM are in an approximately linear range so that investigations with the concentration of either 5 or 10 µM are reasonable. IR-783@BSA, IR-808@BSA, and IR-780@BSA fluorophores were selected to have higher fluorescence enhancement compared with the rest of dye fluorophores (Figure 1C). In particular, IR-780@BSA had a 24-fold fluorescence increase compared to IR-780 in PBS buffer. We next optimized the molar ratio of dye and BSA and found that an equal dye/albumin molar ratio produced the highest brightness for the best three fluorophores (IR-783@BSA, IR-808@BSA, and IR-780@BSA) (Figure 1D, and then all the mentioned ratios in this work were molar ratios). The fluorophore could provide sufficient NIR-II brightness under the longer NIR-II sub-window by increasing the exposure time (Figure S3A). We also excluded the influence of a trace amount of DMSO in the preparation process by comparing the brightness before and after ultrafiltration (Figure S3B). Next, we tested the photostability of all fluorophores with continuous laser irradiation (Figure 1E). Results indicated that the half-life for most dye@BSA fluorophores was less than 10 min. In contrast, IR-780@BSA was exceptional with a long half-life of over 25 min under the NIR-II window, and it was more obvious in the NIR-I region with a half-time of over 40 min (Figure S3C). The bathochromic shift of IR-780@BSA and fluorescence enhancement were potentially the result of confined twisted intramolecular charge transfer (TICT) (Figure 1F, G, and Figure S4) 24, 41, 42. Moreover, the photostabilities of IR-783, IR-808, and IR-780 in DMSO were almost the same, conversely, the brightness and photostability of IR-780 in PBS decreased due to its hydrophobicity. The photostability of IR-780@BSA was dramatically improved, indicating that binding with BSA greatly improved the photostability of IR-780 (Figure S5).

Although the optimized condition could produce stable dye@albumin fluorophores for IR-780, IR-783, and IR-808 (Figure S1D), we found that IR-808 and IR-783 need a longer time to reach a complete reaction compared with IR-780 36. This result was consistent with the photostability sequence of the fluorophores: IR-780@BSA > IR-808@BSA > IR-783@BSA. To further investigate the interaction mechanism between dyes and albumin, we also manufactured a set of IR-780@BSA fluorophores with proportionally increasing molar ratios of IR-780 and subjected them to continuous laser exposure (Figure S6A, B). A “declining” curve of fluorescence brightness versus time was obtained when the ratio of dye to BSA was less than 1, while if the ratio was greater than 1, the “parabola” brightness-variation curve was observed. As the dye/BSA ratio was increased from 1 to 16, it would adjust the peak brightness from 0 to 25 min. We hypothesized that the redundant free dyes caused intramolecular quenching, and the laser exposure would preferentially destroy the free dyes as the cyanine@BSA fluorophore would efficiently protect the bound dyes (Figure S6C). As a result, the brightness curves from different ratios were consistent after the brightness declined gradually. It appears that the redundant free dyes only quenched the brightness of cyanine@BSA fluorophores but did not change their inherent properties.

### 2.2. Investigating the mechanism of super photostability of IR-780@BSA fluorophore

**Figure 2:**
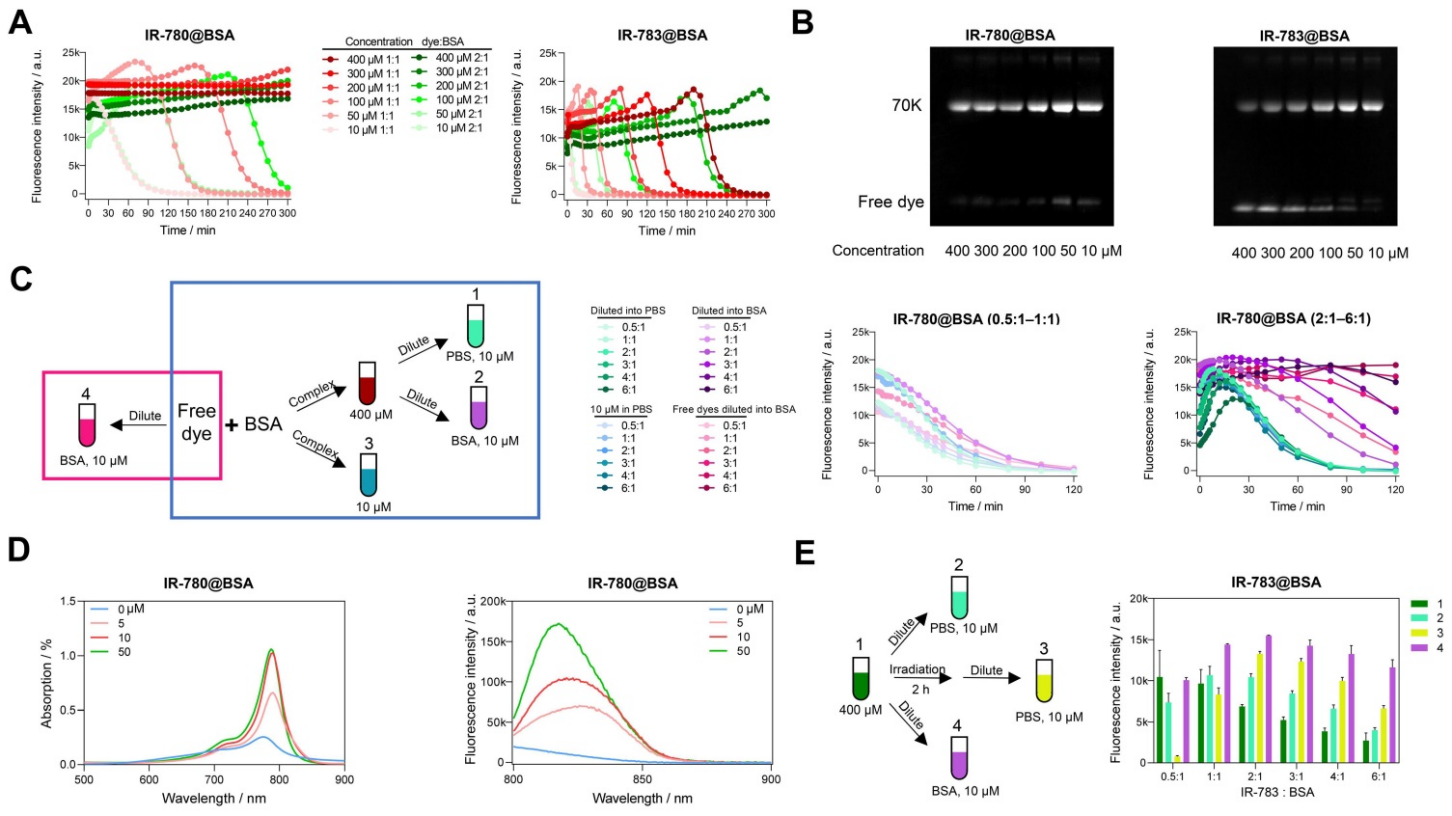
** Deviation of optical performances of IR-780@BSA and IR-783@BSA under different conditions.** Photostability (A) and electrophoresis analysis (B) of IR-780@BSA and IR-783@BSA (1:1 and 2:1 ratios) with reaction concentration from 400 to 10 μM. (C) Simplified schematic diagram and photostability of IR-780@BSA fluorophores obtained under different conditions (400 μM cyanine@BSA fluorophores diluted into PBS (10 μM, Group 1) and 50 mg/mL BSA (10 μM, Group 2), and original 10 μM reaction system (10 μM, Group 3), as well as free dye diluted into 50 mg/mL BSA (10 μM, Group 4)) with reaction/mixing ratio from 0.5:1 to 6:1 (dye:BSA). (D) The absorption and emission spectra of IR-780 (10 μM) in PBS after adding BSA of different concentrations (0, 5, 10, and 50 μM). (E) Simplified schematic diagram and brightness of another four groups of IR-783@BSA fluorophores: original 400 μM reaction system (400 μM, Group 1), 400 μM reaction system diluted into PBS (10 μM, Group 2), 400 μM reaction system irradiated for 2 h and then diluted into PBS (10 μM, Group 3), and 400 μM reaction system diluted into BSA (10 μM, Group 4) with reaction/mixing ratio from 0.5:1 to 6:1 (dye:BSA). All fluorescence intensity data and NIR-II images were performed with 900+1000 nm long-pass filters under 808 nm laser irradiation (60 mW/cm^2^).

To comprehensively understand why IR-780@BSA was much more stable than IR-783@BSA, we further investigated the photostability of IR-780@BSA/IR-783@BSA fluorophores with tunable reaction concentrations and ratios. The cyanine@BSA fluorophores with higher concentrations were more tolerant to laser exposure (Figure 2A, B), while IR-780@BSA was more stable than IR-783@BSA within all tested reaction concentrations. For example, the timepoint of brightness decay for IR-780@BSA and IR-783@BSA (100 μM concentration with 1:1 ratio) occurred at 160 and 35 min, respectively. We further selected a group of three combinations: dye:BSA = 1:1 (100 μM), dye:BSA = 2:1 (100 μM), and dye:BSA = 1:1 (200 μM), and compared the timepoint of brightness decay (t). The sequence was t_100, 1:1_ < t_100, 2:1_ < t_200, 1:1_, while the brightness (B) sequence was B_100, 1:1_ > B_100, 2:1_. Collectively, the photostability was mainly determined by the concentration of cyanine@BSA fluorophores, and the excess dyes would quench the brightness of cyanine@BSA fluorophore and extend the timepoint of brightness decay.

We next explored the photostability of cyanine@BSA system from high concentration (400 μM) diluting into low concentration (10 μM) with PBS and 50 mg/mL BSA to simulate the blood environment (Group 1, 2 in Figure 2C). The photostability tendency was similar in PBS and BSA when the dye to protein ratio was less than 1, indicating that the cyanine@BSA fluorophore was not influenced by the buffer environment when the dye was combined with BSA completely (Figure 2C, middle). We discovered that the extra free dyes in cases of cyanine@BSA system with molar ratio > 1 would quench the fluorophore brightness while pure cyanine@BSA could preserve the initial brightness (Figure 2C, right), thus BSA buffer could greatly improve the brightness of the cyanine@BSA system (ratio > 1) by combining with free dyes. And the similar tendency between diluted (Group 1) and original 10 μM cyanine@BSA system (Group 3) proved that concentration could affect photostability but not change the binding properties. For the cyanine@BSA system with a molar ratio > 1, the diluted fluorophore in BSA buffer (Group 2) was much more photostable than both the diluted fluorophore in PBS (Group 1) and the original 10 μM reaction system (Group 3). Interestingly, samples with free IR-780 diluted in BSA (Group 4) displayed similar photostability compared with the pre-synthesized IR-780@BSA fluorophore in BSA buffer (Group 2). Collectively, we reasonably speculated the high affinity between IR-780 and BSA provided the ability to be protected by albumin (forming covalent fluorophore) even at room temperature or under physiological conditions 36. Besides, the absorption and emission spectra of IR-780 in different concentrations of BSA showed that the brightness was dominated by the interaction between IR-780 and BSA. Although the peak emission (800-850 nm) increased with extra BSA, the intensity of tail emissions over 850 nm were equivalent when BSA to IR-780 ratio was no less than 1 (Figure 2D).

We additionally compared the brightness of the high concentration (400 μM) cyanine@BSA fluorophores with 120 min irradiation before and after diluting into PBS (Figure 2E). The brightness of IR-780@BSA of irradiated samples was nearly equal to the non-irradiated ones for all tested ratios due to its high photostability (Figure S7A). In contrast, the brightness of IR-783@BSA after irradiation declined for dye:BSA ratio < 1, while increased for dye:BSA ratio > 1 (Group 3 in Figure 2E). This result elaborated that IR-780@BSA was much more stable than IR-783@BSA, and irradiation could partially damage both free dyes and dyes in IR-783@BSA (400 μM system). The diluted fluorophores (10 μM, PBS buffer) could partially enhance the brightness for IR-783@BSA system with dye:BSA ratio > 1 due to the presence of extra free IR-783 originally quenched in PBS (Group 2 in Figure 2E). The irradiation further broke the free IR-783 in IR-783@BSA system (Group 3 in Figure 2E), which decreased the quenching effect of free IR-783 for IR-783@BSA and thus improved the brightness of Group 3. In addition, when diluting into BSA buffer, the free IR-783 could combine with excess BSA to generate new cyanine@BSA fluorophores, thus the brightness was further enhanced (Group 4 in Figure 2E).

### 2.3. The biding sites between cyanine dyes and albumin domains

**Figure 3:**
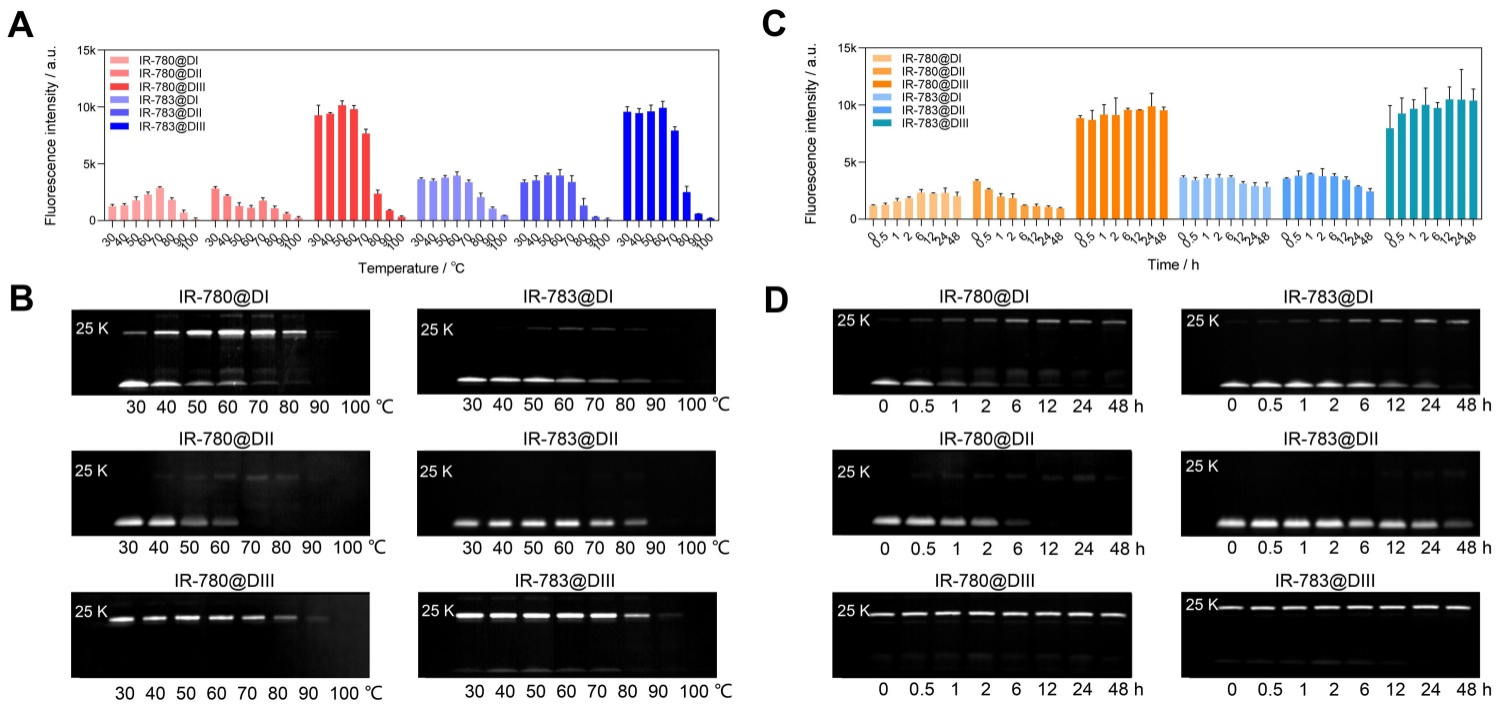
** The brightness and interaction between IR-780/IR-783 and albumin domains.** Brightness and electrophoresis analysis of IR-780@Domains and IR-783@Domains with reaction temperature (A, B), reaction time (C, D) optimization under 808 nm laser irradiation (60 mW/cm^2^) with 900/1000 nm long-pass filters. The concentration of all tested samples was 4 μM.

We have experimentally confirmed that the covalent bond was formed between chloride-containing dyes and albumin/albumin fragments. We further investigated the binding ability between IR-780/IR-783 and albumin domain I (DI), domain II (DII), and domain III (DIII) 43, 44. From brightness data (Figure 3A, C), dye@DIII was much brighter than dye@DI and dye@DII fluorophores/mixtures. The brightness of dye@DIII fluorophores dramatically declined when the reaction temperature was over 70 ºC. Interestingly, higher temperatures and longer reaction time could gradually improve the brightness of IR-780@DI. From the electrophoretic results (Figure 3B, D), both DI and DIII could effectively form the covalent bonds with cyanine dyes. Chloride-containing cyanine dyes could covalently bind with DIII even at mild temperatures (*e.g.* 30 ºC) and short reaction time (*e.g.* few minutes), while these dyes could only covalently bind with DI at higher temperatures (*e.g.* >30 ºC) and longer reaction time (*e.g.* few hours). Unfortunately, the covalent binding did not enhance the brightness of the dye@DI fluorophores, perhaps due to the lack of supramolecular confinement. From the spectra data (Figure 1F, G, and Figure S4C-F), DI and DIII displayed the same effect as BSA that promoted the absorption peak bathochromic-shift, but only DIII enhanced the absorption and emission intensity simultaneously. We next manufactured a set of IR-780@DIII fluorophores with different dye-to-DIII ratios and examined the photostability under continuous 808 nm laser irradiation (Figure S6D). Similar brightness-variation curves were observed for dye@BSA fluorophores, however, the photostability of dye@DIII was worse than dye@BSA due to insufficient protection of protein pocket and/or supramolecular restriction (Figure S6E).

### 2.4. The molecule structures govern the covalent binding to albumin/albumin fragments

**Figure 4:**
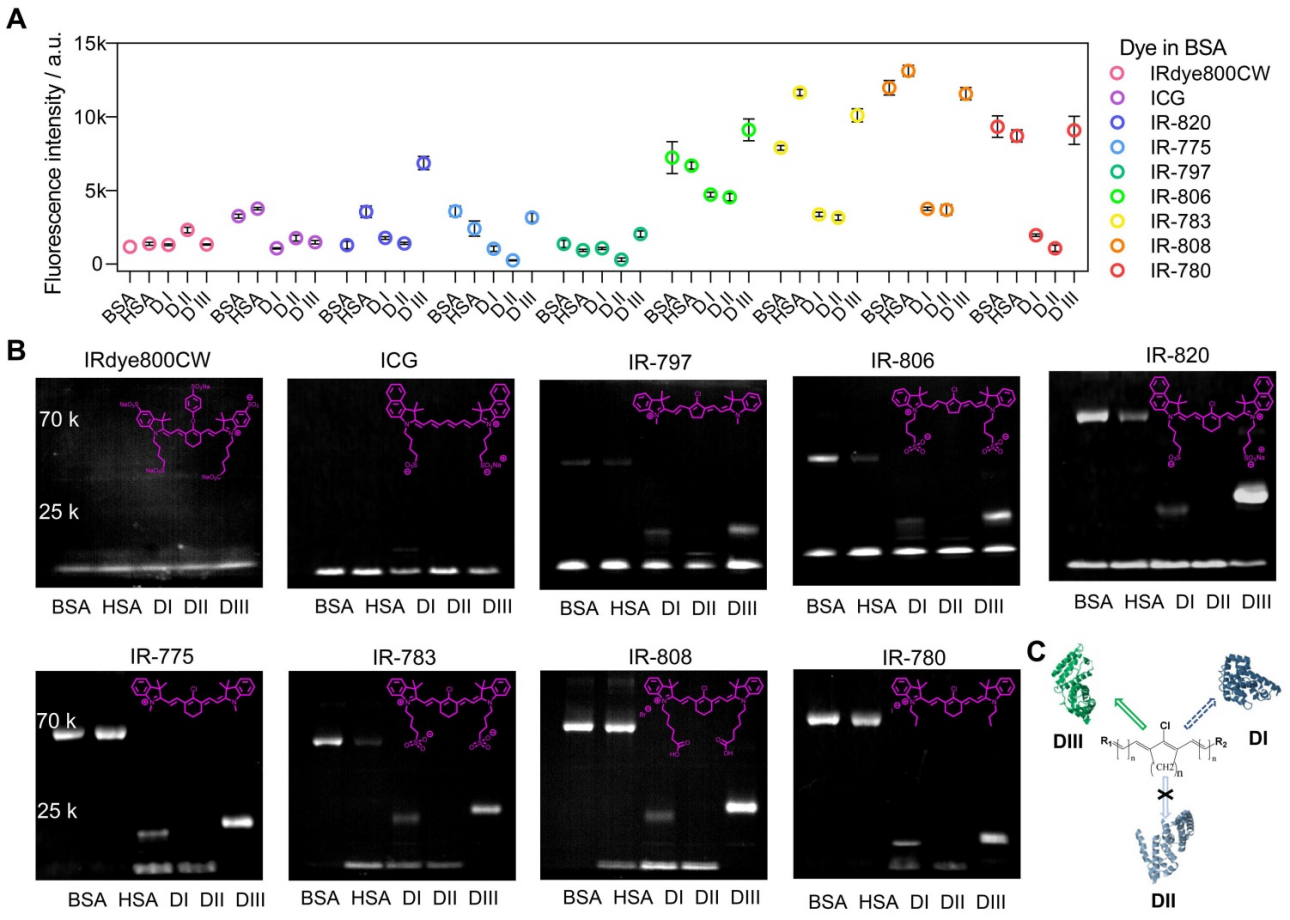
** The influence of interactions between dye structure and albumin/albumin fragments.** The fluorescence brightness (A) and electrophoresis analysis (B) of dye interacting with BSA, HSA, DI, DII, and DIII. The concentration of samples was 4 μM. (C) The diagram of forming a covalent bond between cyanine dye and albumin domains.

To accurately understand the interaction between cyanine dyes and albumin/albumin fragments, we systematically analyzed the covalent binding ability using commercially available cyanine dyes (ICG, IRdye800CW, IR-775, IR-780, IR-783, IR-797, IR-806, IR-808, and IR-820) through the cyanine@proteins brightness inspection and electrophoresis analysis (Figure 4A, B) 45. Here we chose BSA, human serum albumin (HSA), and albumin DI, DII, and DIII to survey the fluorophore brightness and covalent binding of dyes to albumin fragments. Our previous study has adopted super high-resolution liquid chromatography with mass spectrometry (LC-MS/MS) to confirm that the covalently bound cyanine@proteins could be characterized by electrophoresis analysis. Comparing the brightness data of nine sets of cyanine@proteins (Figure 4A), the brightness enhancement of dye@protein was not dependent on the covalent binding. DIII was the major binding site while DI was the secondary binding site (Figure 4C). However, the covalent binding between dyes and DI did not improve the brightness. Taken together, efficient supramolecular interaction between dye and protein is essential to immobilize the TICT conformation, thus enhancing the NIR-II brightness 24, 41, 46.

From the electrophoresis analysis (Figure 4B), cyanine dyes possessing chlorocyclohexene groups such as IR-775, IR-780, IR-783, and IR-808 almost had complete interaction with BSA, HSA, and DIII while the IR-820 was an exception. Hence, we reasonably speculated the steric hindrance of naphthalene groups prevented the sufficient approach between albumin and IR-820, thus reducing the percentage of covalent binding for a limited heating time. This hypothesis was further confirmed by the relatively high extent of interaction of IR-820 with DIII, in which the steric hindrance was not an issue for IR-820 to approach the binding site of “small” DIII. Furthermore, the subset of Cl-free dyes such as IRdye800CW and ICG did not observe covalent bonds with protein in electrophoresis analysis. A noteworthy point of this subset was that IRdye800CW almost didn't increase fluorescence at all with any proteins. This point allowed us to assume that the steric hindrance and hydrophilicity prevented the efficient supramolecular interaction. The ICG showed brightness enhancement when mixing with BSA or HSA, but no brightness enhancement for albumin domains. This result further indicated that the hydrophobic pocket of the whole albumin was essential to anchor both Cl-containing and Cl-free dyes, while the DIII needs covalent binding to efficiently “hook” the Cl-containing dyes. We next compared the final subset of Cl-containing dyes including five-membered ring (IR-797, IR-806) and six-membered ring (IR-775, IR-783) pairs, and we found cyanine dyes with the five-membered ring only formed partial covalent binding compared with six-membered-ring dyes.

### 2.5. Docking modeling revealed interaction discrepancy between cyanine dye and albumin

**Figure 5:**
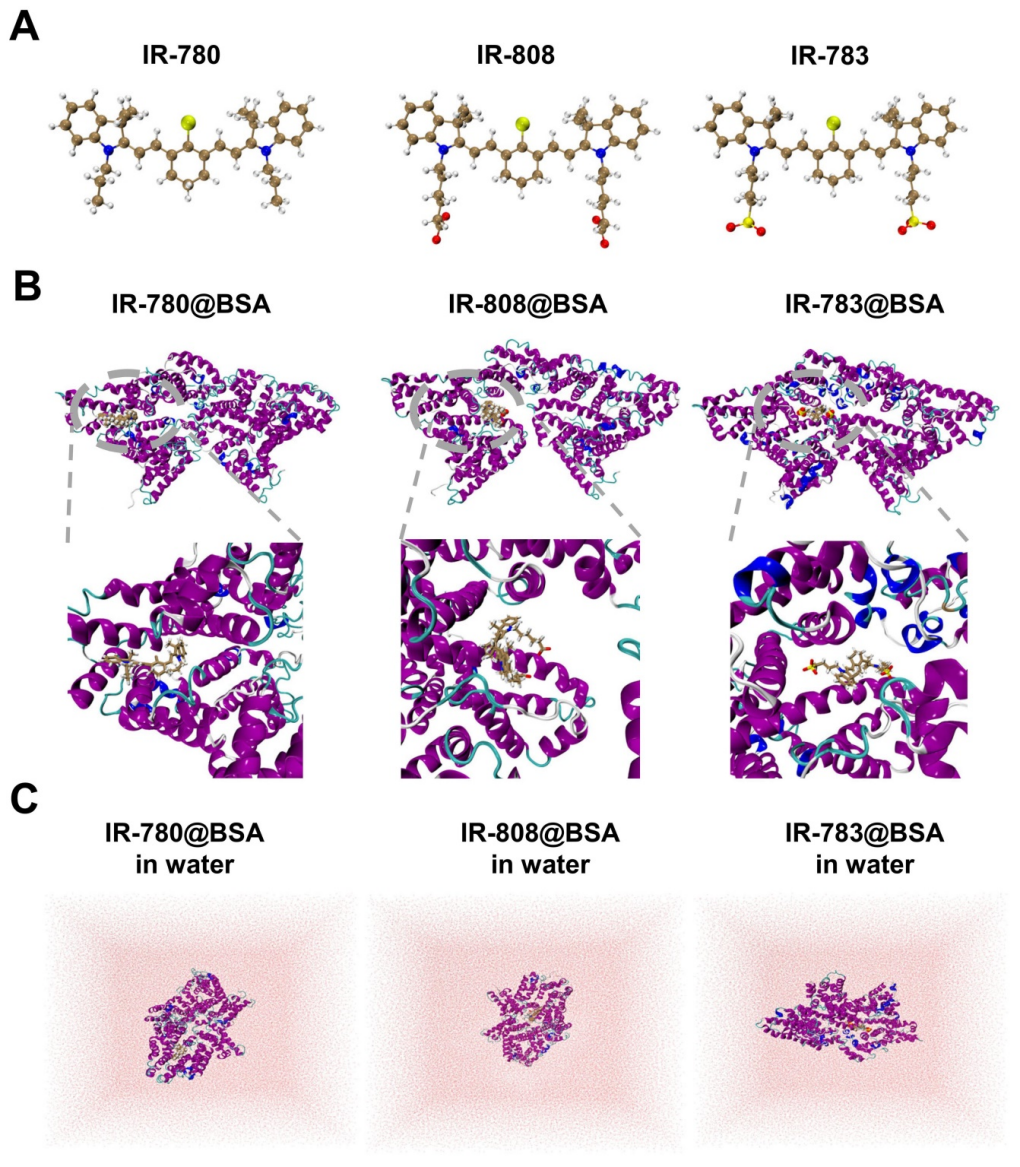
** Docking modeling of cyanine@BSA fluorophores.** (A) The optimized geometries of IR-780, IR-808, and IR-783 molecules by DFT at the tuned LC-BLYP*/6-311+G(d) level. (B) Binding models of the corresponding cyanine@BSA fluorophores. (C) The molecular dynamics simulation of the three molecules in water.

Investigating the interaction (photostability) discrepancy of IR-780, IR-808, and IR-783 with albumin is critical to understanding the binding mechanism and guiding the efficient synthesis of proper fluorescent dyes. To study the binding interactions between BSA protein and 780, 808, 783 ligands, molecular docking modeling was performed and the promising binding modes were further confirmed by long-time molecular dynamics (MD) simulations (see Figure 5 and Supplementary Material for the computational details). It could be seen that all three protein-ligand fluorophores reached equilibrium after 150 ns simulation (Figure S8). As shown in Figure 5, the binding sites of IR-783@BSA and IR-808@BSA are similar, but the binding site of IR-780@BSA seems to be different with a tighter package compared to the other two fluorophores, indicating stronger binding stability. To further semi-quantitatively characterize the stability of these three protein-ligand fluorophores, i.e., IR-780@BSA, IR-808@BSA, and IR-783@BSA, a post-processing end-state method-molecular mechanics/generalized Born surface area (MM/GBSA) 47, 48 based on 10000 configurations taken from the equilibrated MD trajectories were performed to calculate their binding free energies in water (Figure 5C and Table S2). The MM/GBSA method is known to be one of the most popular methods to estimate binding free energies since it achieves a good balance between accuracy and computational efficiency, compared to other methods such as free energy perturbation (FEP) 49 and thermodynamic integration (TI) 50 methods. Thus, the relative binding free energies calculated by MM/GBSA method for these three protein-ligand fluorophores were -47.56, -42.13, and -36.14 kcal/mol, respectively, which agreed with the trend of observed experimental photostability. Also, a significantly negative ΔG_gas_ value of -249.46 kcal/mol for IR-780@BSA also suggests IR-780 is more conducive to combine with BSA in the gas phase (Table S2). Compared to IR-808 and IR-783, IR-780 shows relatively positive charge distribution (Figure S9), indicating its preferable interaction with albumin and binding free energies. The high hydrophobicity feature of IR-780 also promotes the interaction with the hydrophobic pocket of BSA.

### 2.6. The binding feature of dye@protein fluorophores determines their *in vivo* pharmacokinetics

**Figure 6:**
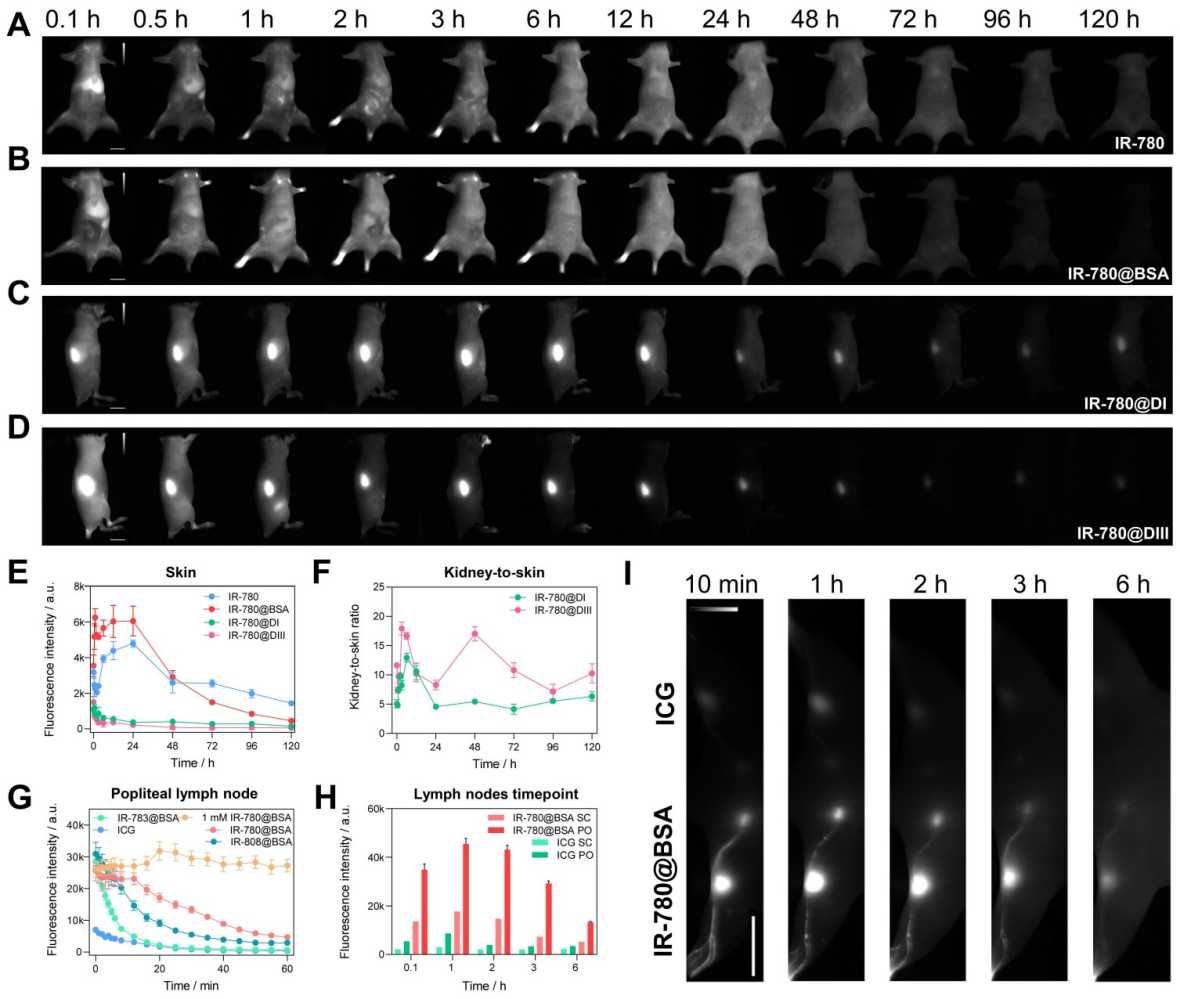
** The comparison of metabolism behavior for IR-780 and its albumin/albumin domain fluorophores, and IR-780@BSA enables long-lasting lymph node imaging.** Whole-body imaging with (A) IR-780, (B) IR-780@BSA, (C) IR-780@DI, and (D) IR-780@DIII (150 μM, 200 μL) as a function of injected time points. Exposure time was 20 ms. The scale bar was 1 cm. (E) The skin (or skin + muscle) brightness intensity profile of IR-780, IR-780@BSA, IR-780@DI, and IR-780@DIII. (F) The kidney-to-skin ratio of IR-780@DI and IR-780@DIII. (G) The brightness of popliteal lymph node as a function of time with intradermal injection of IR-780@BSA, IR-783@BSA, IR-808@BSA, and ICG at equivalent dosages (150 μM, 25 μL) under the continuous irradiation. Notably, a higher dosage of 1 mM IR-780@BSA (25 μL) provided a super-stable lymph node imaging without obvious signal decay up to 60 min. (H-I) The comparison of lymph nodes imaging with clinically-available ICG and IR-780@BSA fluorophore. The scale bar was 4 cm. All NIR-II images were captured with 1000 /1100 nm long-pass filters under 808 nm laser irradiation (60 mW/cm^2^). The fluorescence intensity scale bar of A-D and I ranges from 0-12000, 0-12000, 0-4000, 0-4000, and 0-30000, respectively.

The findings in the diversity of dye@protein binding prompted us to assess the fundamental metabolism of fluorophores (Figure 6A-D and Figure S10). After intravenous administration of IR-780@BSA, it quickly accumulated in the liver and then distributed throughout the whole body, followed by faeces excretion, sharing a similar hepatobiliary excretion pathway with free IR-780 (Figure 6A, B and Figure S11D). In addition, rather high brightness was found from the liver and intestines at 24 h timepoint under* ex vivo* fluorescence imaging, which further proves the hepatobiliary excretion pathway (Figure S12). Because of its highest binding affinity (t_1/2_ = 2 min at 37 ^o^C, binding completely within an hour) 36 among IR-780, IR-808, and IR-783, free IR-780 could quickly form a similar IR-780@albumin with endogenous albumin in the body 36. In addition, endogenous albumin was also enough for IR-808 (t_1/2_ = 6 h at 37 ^o^C, binding completely after reacting for 72 h) 36 binding after injecting free IR-808 into the mice, thus its metabolic behavior was similar to IR-808@BSA and IR-780@BSA (Figure S10A, B). In contrast, due to the relatively weak binding affinity of IR-783 (t_1/2_ = 72 h at 37 ^o^C, ~60 % free dye was left after reacting for 72 h) 36 with albumin, free IR-783 cleared rapidly from the body within 24 h (Figure S10C) 24. This differs distinctly from the IR-783@BSA fluorophore, which exhibited similar metabolism behavior to IR-780@BSA (Figure S10D and Figure S11).

We then assessed the *in vivo* behavior of IR-780@DI and IR-780@DIII. Both domain-derived fluorophores exhibited the renal excretion pathway due to the much smaller size (Figure 6C, D and Figure S13A, B). A noteworthy feature was that the body skin (or skin + muscle) signal of IR-780@DI was higher than that of IR-780@DIII, again proving the relatively weak binding between IR-780 and DI. The dropping free dyes from IR-780@DI further combined with endogenous albumin, thus increasing the skin/muscle signal (Figure 6E). Although kidney signal was not much different for IR-780@DI and IR-780@DIII administered mice (Figure S13C), the kidney-to-skin ratio of IR-780@DIII was more prominent from 24 to 96 h time points (Figure 6F and Figure S13D).

We subsequently traced the *in vivo* behavior of different dye@BSA fluorophores and proved that the metabolic behavior of fluorophores was related to the degree of covalent bonding. First, Cl-free dye@albumin fluorophore displayed rapid hepatobiliary elimination, similar to the free dye (see ICG@BSA case in Figure S10E). Second, the metabolic time of Cl-containing dye@albumin fluorophores was consistent with the photostability results (IR-780@BSA > IR-808@BSA > IR-783@BSA) (Figure 6B, Figure S10B, D and Figure S11C). In other words, the *in vivo* metabolic behavior could indirectly reflect the photostability feature of the dye@BSA fluorophore. At last, IR-820@BSA with partial covalent binding exhibited two metabolic forms of both free IR-820 and IR-820@BSA (Figure S10F) 25.

After proving the high photostability and metabolic behavior of IR-780@BSA, we turned to performing lymph node imaging to assess their NIR-II imaging ability 27. We investigated the photostability of lymph node imaging using three dye@BSA fluorophores (IR-780@BSA, IR-808@BSA, IR-783@BSA) and clinically-available ICG. IR-780@BSA was still most stable among the four tested probes (photostability sequence: IR-780@BSA > IR-808@BSA > IR-783@BSA > ICG) under the continuous laser irradiation (Figure 6G). Notably, the elevated injection dosage of IR-780@BSA (1 mM, 25 μL) provided a photostable NIR-II imaging window for popliteal lymph nodes visualization, and no signal decay was observed for up to 60-min continuous laser-irradiation (Figure 6G). The popliteal and sacral lymph nodes were clearly outlined in a prone position with IR-780@BSA intradermally injected in the left footpad and ICG injected in the right as a clinically-used reference. The imaging window was tested for up to 6 h (lymph node-to-skin ratio ≥ 3.6), which was an essential feature for the long-term imaging-guided surgery process (Figure 6H, I).

## 3. Discussion and Conclusion

Albumin is the most abundant protein in the blood of living organisms. Studies have repeatedly shown that albumin could be used as an efficient drug/dye carrier to prolong their *in vivo* circulation time 51-53. An in-depth study of the interaction between albumin and dyes is essential to identify binding mechanisms and develop improved imaging agents. Previous reports proved that the flexible cyanine dye could be lodged and stabilized by albumin scaffold through covalent bonding, enhancing the twisted intramolecular charge transfer process 24, 41, 46. We systematically substantiated that the covalently bound dyes to albumin/fragments effectively prolonged the imaging window or pharmacokinetics. Notably, we found that the more stable covalent combination formed between cyanine dye and proteins, the better photostability of bioimaging was obtained.

The current study clarified the detailed binding feature through several sets of dye and albumin/albumin fragment fluorophores, providing the general principle to design either albumin-chaperoned or albumin-escaping dyes. The cyanine dyes which can be favorably lodged into albumin represent several appropriate features in terms of a six-membered ring with chlorine, the small size of side groups, and relatively high hydrophobicity. Cyanine dyes with different structures (IR-780 and IR-783 are two extreme examples) can have entirely different binding rates/strengths. As the formation process of dye@protein fluorophore contains two steps including supramolecular assembly and covalent hooking, reaction temperature/time can facilitate the supramolecular interaction and covalent binding between dyes and proteins 54. For example, the reaction solution of IR-783@BSA could get rid of the free IR-783 when increasing the temperature from 37 to 50 ^o^C for two hours. The hydrophobic dye IR-780 can form a stable covalent bond with albumin within a few minutes, but the brightness of the fluorophore increases with the extension of heating. We presumed that continuous heating promotes the hydrophobic dye to conformation-preferentially lodge into the hydrophobic cavity of albumin, further decreasing the internal rotation/vibration transition. Additionally, we speculated that small organic molecules could have the ability to bind to proteins if they have favorable steric hindrance and reactive sites with proteins. Much effort has been made to conquer the protein crown issue of small molecular drugs and/or imaging agents. Our investigation sheds light on the design principle of cyanine dyes with less interaction with proteins and affords reasonable support for achieving albumin-escaping dyes 46, 55. Especially, when the binding between dye and albumin is slow enough, it can be considered as a non-covalent combination and this dye is a potential “albumin-escaping” agent. Collectively, our findings provide a rational framework for the enrichment of the NIR-II fluorescent probe library and promoting the clinical translation of NIR-II bioimaging 56-58.

## Supplementary Material

Supplementary experimental section, figures and tables.

## Abbreviations

NIR-II: Near-infrared-II
Cl: chlorine
QY: quantum yield
IR-780@BSA: IR-780@bovine serum albumin
ACQ: aggregation caused quench
TICT: twisted intramolecular charge transfer
DI: albumin domain I
DII: albumin domain II
DIII: albumin domain III
HSA: human serum albumin
MD: molecular dynamics
MM/GBSA: method-molecular mechanics/generalized Born surface area
FEP: free energy perturbation
TI: thermodynamic integration

## References

[1] Miao Q, Pu K (2018). Organic Semiconducting Agents for Deep-Tissue Molecular Imaging: Second Near-Infrared Fluorescence, Self-Luminescence, and Photoacoustics. Adv Mater.

[2] Hong G, Lee JC, Robinson JT, Raaz U, Xie L, Huang NF (2012). Multifunctional in vivo vascular imaging using near-infrared II fluorescence. Nat Med.

[3] Kenry Duan Y, Liu B (2018). Recent Advances of Optical Imaging in the Second Near-Infrared Window. Adv Mater.

[4] Welsher K, Liu Z, Sherlock SP, Robinson JT, Chen Z, Daranciang D (2009). A route to brightly fluorescent carbon nanotubes for near-infrared imaging in mice. Nat Nanotechnol.

[5] Zhu S, Tian R, Antaris AL, Chen X, Dai H (2019). Near-Infrared-II Molecular Dyes for Cancer Imaging and Surgery. Adv Mater.

[6] Hu Z, Fang C, Li B, Zhang Z, Cao C, Cai M (2020). First-in-human liver-tumour surgery guided by multispectral fluorescence imaging in the visible and near-infrared-I/II windows. Nat Biomed Eng.

[7] Zhu S, Yung BC, Chandra S, Niu G, Antaris AL, Chen X (2018). Near-Infrared-II (NIR-II) Bioimaging via Off-Peak NIR-I Fluorescence Emission. Theranostics.

[8] Hong G, Antaris AL, Dai H (2017). Near-infrared fluorophores for biomedical imaging. Nat Biomed Eng.

[9] Qi J, Sun C, Zebibula A, Zhang H, Kwok RTK, Zhao X (2018). Real-Time and High-Resolution Bioimaging with Bright Aggregation-Induced Emission Dots in Short-Wave Infrared Region. Adv Mater.

[10] Cai R, Chen C (2019). The Crown and the Scepter: Roles of the Protein Corona in Nanomedicine. Adv Mater.

[11] Madathiparambil Visalakshan R, Gonzalez Garcia LE, Benzigar MR, Ghazaryan A, Simon J, Mierczynska-Vasilev A (2020). The Influence of Nanoparticle Shape on Protein Corona Formation. Small.

[12] Yang ST, Liu Y, Wang YW, Cao A (2013). Biosafety and bioapplication of nanomaterials by designing protein-nanoparticle interactions. Small.

[13] Tenzer S, Docter D, Kuharev J, Musyanovych A, Fetz V, Hecht R (2013). Rapid formation of plasma protein corona critically affects nanoparticle pathophysiology. Nat Nanotechnol.

[14] Yu M, Zheng J (2015). Clearance Pathways and Tumor Targeting of Imaging Nanoparticles. ACS Nano.

[15] Wang B, He X, Zhang Z, Zhao Y, Feng W (2013). Metabolism of Nanomaterials in Vivo: Blood Circulation and Organ Clearance. Acc Chem Res.

[16] Poon W, Zhang Y-N, Ouyang B, Kingston BR, Wu JLY, Wilhelm S (2019). Elimination Pathways of Nanoparticles. ACS Nano.

[17] Cheng P, Pu K (2021). Molecular imaging and disease theranostics with renal-clearable optical agents. Nat Rev Mater.

[18] Lee ES, Youn YS (2016). Albumin-based potential drugs: focus on half-life extension and nanoparticle preparation. J Pharm Investig.

[19] An FF, Zhang XH (2017). Strategies for Preparing Albumin-based Nanoparticles for Multifunctional Bioimaging and Drug Delivery. Theranostics.

[20] Liu Z, Chen X (2016). Simple bioconjugate chemistry serves great clinical advances: albumin as a versatile platform for diagnosis and precision therapy. Chem Soc Rev.

[21] Sheng Z, Hu D, Zheng M, Zhao P, Liu H, Gao D (2014). Smart Human Serum Albumin-Indocyanine Green Nanoparticles Generated by Programmed Assembly for Dual-Modal Imaging-Guided Cancer Synergistic Phototherapy. ACS Nano.

[22] Chen Q, Liang C, Wang C, Liu Z (2015). An imagable and photothermal "Abraxane-like" nanodrug for combination cancer therapy to treat subcutaneous and metastatic breast tumors. Adv Mater.

[23] Tian L, Shao M, Gong Y, Chao Y, Wei T, Yang K (2022). Albumin-binding lipid-aptamer conjugates for cancer immunoimaging and immunotherapy. Sci China Chem.

[24] Tian R, Zeng Q, Zhu S, Lau J, Chandra S, Ertsey R (2019). Albumin-chaperoned cyanine dye yields superbright NIR-II fluorophore with enhanced pharmacokinetics. Sci Adv.

[25] Feng Z, Yu X, Jiang M, Zhu L, Zhang Y, Yang W (2019). Excretable IR-820 for in vivo NIR-II fluorescence cerebrovascular imaging and photothermal therapy of subcutaneous tumor. Theranostics.

[26] Du B, Qu C, Qian K, Ren Y, Li Y, Cui X (2019). An IR820 Dye-Protein Complex for Second Near-Infrared Window and Photoacoustic Imaging. Adv Opt Mater.

[27] Antaris AL, Chen H, Diao S, Ma Z, Zhang Z, Zhu S (2017). A high quantum yield molecule-protein complex fluorophore for near-infrared II imaging. Nat Commun.

[28] Zeng X, Xiao Y, Lin J, Li S, Zhou H, Nong J (2018). Near-Infrared II Dye-Protein Complex for Biomedical Imaging and Imaging-Guided Photothermal Therapy. Adv Healthc Mater.

[29] Li B, Lu L, Zhao M, Lei Z, Zhang F (2018). An Efficient 1064 nm NIR-II Excitation Fluorescent Molecular Dye for Deep-Tissue High-Resolution Dynamic Bioimaging. Angew Chem, Int Ed.

[30] Li D, Qu C, Liu Q, Wu Y, Hu X, Qian K (2020). Monitoring the Real-Time Circulatory System-Related Physiological and Pathological Processes In Vivo Using a Multifunctional NIR-II Probe. Adv Funct Mater.

[31] Cosco ED, Spearman AL, Ramakrishnan S, Lingg JGP, Saccomano M, Pengshung M (2020). Shortwave infrared polymethine fluorophores matched to excitation lasers enable non-invasive, multicolour in vivo imaging in real time. Nat Chem.

[32] Du Y, Liu X, Zhu S (2021). Near-Infrared-II Cyanine/Polymethine Dyes, Current State and Perspective. Front Chem.

[33] Canovas C, Bellaye P-S, Moreau M, Romieu A, Denat F, Goncalves V (2018). Site-specific near-infrared fluorescent labelling of proteins on cysteine residues with meso-chloro-substituted heptamethine cyanine dyes. Org Biomol Chem.

[34] Usama SM, Lin C-M, Burgess K (2018). On the Mechanisms of Uptake of Tumor-Seeking Cyanine Dyes. Bioconjugate Chem.

[35] Thavornpradit S, Usama SM, Park GK, Shrestha JP, Nomura S, Baek Y (2019). QuatCy: A Heptamethine Cyanine Modification With Improved Characteristics. Theranostics.

[36] Usama SM, Park GK, Nomura S, Baek Y, Choi HS, Burgess K (2020). Role of Albumin in Accumulation and Persistence of Tumor-Seeking Cyanine Dyes. Bioconjugate Chem.

[37] Carr JA, Franke D, Caram JR, Perkinson CF, Saif M, Askoxylakis V (2018). Shortwave infrared fluorescence imaging with the clinically approved near-infrared dye indocyanine green. Proc Natl Acad Sci U S A.

[38] Zhu S, Hu Z, Tian R, Yung BC, Yang Q, Zhao S Repurposing Cyanine NIR-I Dyes Accelerates Clinical Translation of Near-Infrared-II (NIR-II) Bioimaging. Adv Mater. 2018: e1802546.

[39] Tu L, Xie Y, Li Z, Tang B (2021). Aggregation-induced emission: Red and near-infrared organic light-emitting diodes. SmartMat.

[40] Würthner F, Kaiser TE, Saha-Möller CR (2011). J-Aggregates: From Serendipitous Discovery to Supramolecular Engineering of Functional Dye Materials. Angew Chem, Int Ed.

[41] Gao S, Wei G, Zhang S, Zheng B, Xu J, Chen G (2019). Albumin tailoring fluorescence and photothermal conversion effect of near-infrared-II fluorophore with aggregation-induced emission characteristics. Nat Commun.

[42] Zhang Y, Wang S, Wang X, Zan Q, Yu X, Fan L (2021). Monitoring of the decreased mitochondrial viscosity during heat stroke with a mitochondrial AIE probe. Anal Bioanal Chem.

[43] Ghuman J, Zunszain PA, Petitpas I, Bhattacharya AA, Otagiri M, Curry S (2005). Structural Basis of the Drug-binding Specificity of Human Serum Albumin. J Mol Biol.

[44] Sugio S, Kashima A, Mochizuki S, Noda M, Kobayashi K (1999). Crystal structure of human serum albumin at 2.5 Å resolution. Protein Eng Des Sel.

[45] Thavornpradit S, Usama SM, Lin C-M, Burgess K (2019). Protein labelling and albumin binding characteristics of the near-IR Cy7 fluorophore, QuatCy. Org Biomol Chem.

[46] Xing P, Niu Y, Mu R, Wang Z, Xie D, Li H (2020). A pocket-escaping design to prevent the common interference with near-infrared fluorescent probes in vivo. Nat Commun.

[47] Wang E, Sun H, Wang J, Wang Z, Liu H, Zhang JZH (2019). End-Point Binding Free Energy Calculation with MM/PBSA and MM/GBSA: Strategies and Applications in Drug Design. Chem Rev.

[48] Valdés-Tresanco MS, Valdés-Tresanco ME, Valiente PA, Moreno E (2021). gmx_MMPBSA: A New Tool to Perform End-State Free Energy Calculations with GROMACS. J Chem Theory Comput.

[49] Clark AJ, Gindin T, Zhang B, Wang L, Abel R, Murret CS (2017). Free Energy Perturbation Calculation of Relative Binding Free Energy between Broadly Neutralizing Antibodies and the gp120 Glycoprotein of HIV-1. J Mol Biol.

[50] Lawrenz M, Baron R, McCammon JA (2009). Independent-Trajectories Thermodynamic-Integration Free-Energy Changes for Biomolecular Systems: Determinants of H5N1 Avian Influenza Virus Neuraminidase Inhibition by Peramivir. J Chem Theory Comput.

[51] Kramer PA (1974). Letter: Albumin microspheres as vehicles for achieving specificity in drug delivery. J Pharm Sci.

[52] Cho K, Wang X, Nie S, Chen Z, Shin DM (2008). Therapeutic Nanoparticles for Drug Delivery in Cancer. Clin Cancer Res.

[53] Wunder A, Müller-Ladner U, Stelzer EH, Funk J, Neumann E, Stehle G (2003). Albumin-based drug delivery as novel therapeutic approach for rheumatoid arthritis. J Immunol. (Baltimore, Md: 1950).

[54] Shamay Y, Shah J, Isik M, Mizrachi A, Leibold J, Tschaharganeh DF (2018). Quantitative self-assembly prediction yields targeted nanomedicines. Nat Mater.

[55] Choi HS, Gibbs SL, Lee JH, Kim SH, Ashitate Y, Liu F (2013). Targeted zwitterionic near-infrared fluorophores for improved optical imaging. Nat Biotechnol.

[56] Chen Q, Liang C, Wang X, He J, Li Y, Liu Z (2014). An albumin-based theranostic nano-agent for dual-modal imaging guided photothermal therapy to inhibit lymphatic metastasis of cancer post surgery. Biomaterials.

[57] Luo X, Hu D, Gao D, Wang Y, Chen X, Liu X (2021). Metabolizable Near-Infrared-II Nanoprobes for Dynamic Imaging of Deep-Seated Tumor-Associated Macrophages in Pancreatic Cancer. ACS Nano.

[58] Cai Z, Zhu L, Wang M, Roe AW, Xi W, Qian J (2020). NIR-II fluorescence microscopic imaging of cortical vasculature in non-human primates. Theranostics.

