# Supplementary material for: Super-stable cyanine@albumin fluorophore for enhanced NIR-II bioimaging
Source: Theranostics. 2022 May 26;12(10):4536–47. doi: 10.7150/thno.71443 (PMC9254253; doi:10.7150/thno.71443)
Supplement: Supplementary file 1 — Supplementary experimental section, figures and tables. [file thnov12p4536s1.pdf]

Supplementary Material

Super-stable cyanine@albumin fluorophore for enhanced NIR-II bioimaging

Lang Bai, Zhubin Hu, Tianyang Han, Yajun Wang, Jiajun Xu, Guanyu Jiang, Xin Feng, Bin Sun, Xiangping Liu, Rui Tian, Haitao Sun*, Songling Zhang, Xiaoyuan Chen*, Shoujun Zhu**

Experimental section

Materials

ICG was purchased from TCI (Shanghai) Development Co., Ltd. IRdye800CW was purchased from LI-COR Biosciences Co., Ltd. IR-808 was purchased from Shanxi Xinyan Bomei Biotechnology Co., Ltd. Other cyanine dyes were purchased from Sigma-Aldrich. Bovine serum albumin and human serum albumin were purchased from Sigma-Aldrich. Anhydrous dimethyl sulfoxide was purchased from Sigma-Aldrich. Recombinant Human Albumin Domain I, recombinant Human Albumin Domain II, and recombinant Human Albumin Domain III were purchased from Albumin Therapeutics, LLC. PAGE Gel Quick Preparation Kit, GoldBand 3-color Regular Range Protein Marker (10-180 kDa), and 5×SDS-PAGE Protein Loading Buffer were purchased from Yeasen Biotechnology (Shanghai) Co., Ltd.

Cyanine@protein fluorophore

Protein (BSA, HSA) was dissolved in 1 × PBS with a concentration of 10 mg/mL (150 μM). Dye (IR-140, IR800dyeCW, ICG, IR-797, IR-820, IR-775, IR-783, IR-808, IR-780) was dissolved in dimethyl sulfoxide (DMSO) at 5 mM. For a typical reaction, 5 mM of IR-780 was diluted to 500 μM with PBS, and 150 μM of BSA was diluted into 10 μM with PBS. Then, the proportionate dye (500 μM) was added into the BSA solutions, respectively (molar ratios were 0.25:1, 0.5:1, 1:1, 1.25:1, 1.5:1, 1.75:1, 2:1, 3:1, 4:1, 6:1, 8:1, 16:1). For high concentration reaction: BSA was dissolved in 1 × PBS with a concentration of 53.3 mg/mL (~800 μM). Dye (IR-783, IR-780) was dissolved in DMSO at 26.7 mM. For a typical reaction, 800 μM of BSA was diluted to 400, 300, 200, 100, and 50 μM with PBS. Then, the proportionate dye (26.7 mM) was added to the BSA solutions, respectively (the molar ratio was 1:1). Then, the mixed solution was vortexed for 10 seconds and heated at 50 ^{o}C in a shaker for 2 hours. The reaction temperature, reaction concentration, the reaction time were further optimized at different specific steps (see the reaction details in Figure S1 and Figure 1).

The fluorophore can be further purified with Amicon Centrifugal Filter (10-30 kDa) for five times against a PBS buffer. Comparison experiments indicated that trace DMSO did not affect the brightness of the final fluorophore (Figure S3B).

Gel electrophoresis

The fluorophores were loaded into 10 or 15% SDS-PAGE for electrophoresis. The gel was analyzed by detecting the NIR-II fluorescence signal. Then, the gel was stained with Coomassie Brilliant Blue followed by destaining to obtain the band pattern of the various proteins.

Animal experiments.

All animal experiments were conducted under the institutional guidelines and were approved by the Experimental Animal Ethical Committee of Jilin University (protocol number: 20210642). Balb/C mice were purchased from Liaoning Changsheng biotechnology co. Lt. Bedding, nesting materials, food, and water were provided ad libitum. Ambient temperature was controlled at 20 to 24°C with 12-hour light/12-hour dark cycles.

NIR-II imaging

Mice were shaved using Nair hair removal cream and anesthetized using isoflurane. Then tail vein injections were executed with a needle prefilled with imaging agents. The NIR-II set-up was built according to our previous report [1]. In detail, the excitation laser was an 808-nm laser set-up at a power density of 60 mW/cm^2. Emission fluorescence was typically collected with a combination of 1000 and 1100 nm long-pass filters. A tunable exposure time was used for the InGaAs camera to capture images in the NIR-II window. A silicon CCD detector for NIR-I imaging was added in parallel to the NIR-II set-up for NIR-I imaging comparisons.

Statistical Analysis

Unless described otherwise, results are mean ± SD. Two-tailed paired and unpaired Student's t-tests were used to determine differences within groups and between groups, respectively. P values < 0.05 were considered statistically significant.

Spectral characterization

Absorbance spectra of free dyes and fluorophores were acquired on an ultraviolet-visible-NIR PerkinElmer Lambda 950 spectrometer that was background corrected for each media. The NIR-II fluorescence emission spectrum was captured on Edinburgh Instruments FL 920 spectroscopy set-up by exciting with an 808 nm laser. The NIR-I fluorescence emission spectrum was captured on Shimadzu RF-6000 spectroscopy.

Computational Details

For each ligand, the structure was optimized with the Gaussian 16 program [2] at the tuned LC-BLYP* [3]/6-311+G(d) [4-6] level [1, 7, 8] with the polarizable continuum model (PCM) [9] of implicit water solvent, and then the advanced restrained electrostatic potential (RESP2) charges [10, 11] obtained by Multiwfn 3.7 program [12] and the General Amber Force Field (GAFF) [13] got via ACPYPE tool [14] were

assigned for the optimized structure. The structure of BSA protein was taken from RCSB Protein Data Bank [15] (PDB ID: 4F5S) [16] and the AMBER99SB-ILDN [17] force filed was used for protein. The initial structure of protein-ligand fluorophore was obtained by the molecular docking method by AutoDock Vina program [18] and the best docking modes were selected in the following molecular dynamics (MD) simulation. Then, the initial protein-ligand structure was immersed in the center of a 16.5×16.5×16.5 nm^3 cubic box of TIP3P water [19] molecules, and all of the solute atoms were no less than 0.8 nm from the boundary of the water box.

10000 steps of conjugate gradient energy minimizations were carried out to remove bad contacts before simulation. The relaxed structure was then gently heated from 0 to 298.15 K in 100 ps and equilibrated for 100 ps at constant pressure with a position restraint using a harmonic force with a force constant of 1000 $kJ \cdot mol^{-1} \cdot nm^{-2}$ on the protein-ligand fluorophore. And the protein-ligand fluorophore was equilibrated for another 1 ns without restraint. Production simulation in the NPT ensemble was extended to 150 ns with a time step of 2 fs and trajectories were saved every 2 ps. During the simulation, all bonds with hydrogen atoms were fixed using the linear constraint solver (LINCS) algorithm [20]. The particle mesh Ewald (PME) [21] method with an 1 nm cutoff in real space was used to calculate the electrostatic interactions. The cutoff for the nonbonding van der Waals interactions was set to be 1 nm as well. The velocity-rescale thermostat [22] with a coupling time of 0.2 ps was used to regulate temperature. The Berendsen barostat [23] with a time constant of 0.5 ps for the equilibration simulation and the Parrinello-Rahman barostat [24] with a time constant of 2 ps for the production simulation were used to maintain the pressure to 1 bar. All the MD simulations were performed by Gromacs 2020.6 program [25].

After MD simulation, the widely used molecular mechanics/generalized Born surface area (MM/GBSA) [26] method was performed for the calculation of binding free energy of protein-ligand fluorophore. Within MM/GBSA method, the binding free energy can be represented as

$$\langle \Delta G_{bind} \rangle = \langle \Delta H \rangle - \langle T\Delta S \rangle = \langle \Delta E_{gas} \rangle + \langle \Delta G_{sol} \rangle - \langle T\Delta S \rangle.$$

Here, $\langle \ldots \rangle$ means the ensemble average; the ΔG_{bind} is the binding free energy; ΔH is the enthalpy of binding; $-T\Delta S$ is the conformational entropy after ligand binding; ΔE_{gas} is defined as the difference of gas-phase energy between that of the protein−ligand fluorophore and those of the separate protein and ligand, and is computed by MM method; ΔG_{sol} is the difference of solvation free energy between that of the protein−ligand fluorophore and those of the separate protein and ligand systems, which is computed with GBSA method. To compute $\langle \Delta G_{bind} \rangle$, a separate 1 ns MD run with a configurational sampling frequency of 100 fs was performed. Thus, a total of 10000 snapshots were extracted from the MD trajectories for the calculation of binding free energy with the single trajectory protocol. All the MM/GBSA calculations were performed by gmx_MMPBSA tool [27] based on the AMBER's MMPBSA.py tool [28] with default parameters. It should be noted that the entropic term $-T\Delta S$ is not included in our binding free energy and thus the relative

binding free energies correspond to the enthalpy of binding $\langle \Delta H \rangle$. This is a common strategy in the MM/GBSA calculation due to the fact that the entropy calculation is usually very time-consuming, especially for the large system (i.e., the BSA protein with a length of 1166 residues in our case), and enthalpy of binding is usually sufficient for the purpose of comparing relative binding free energies of related ligands.

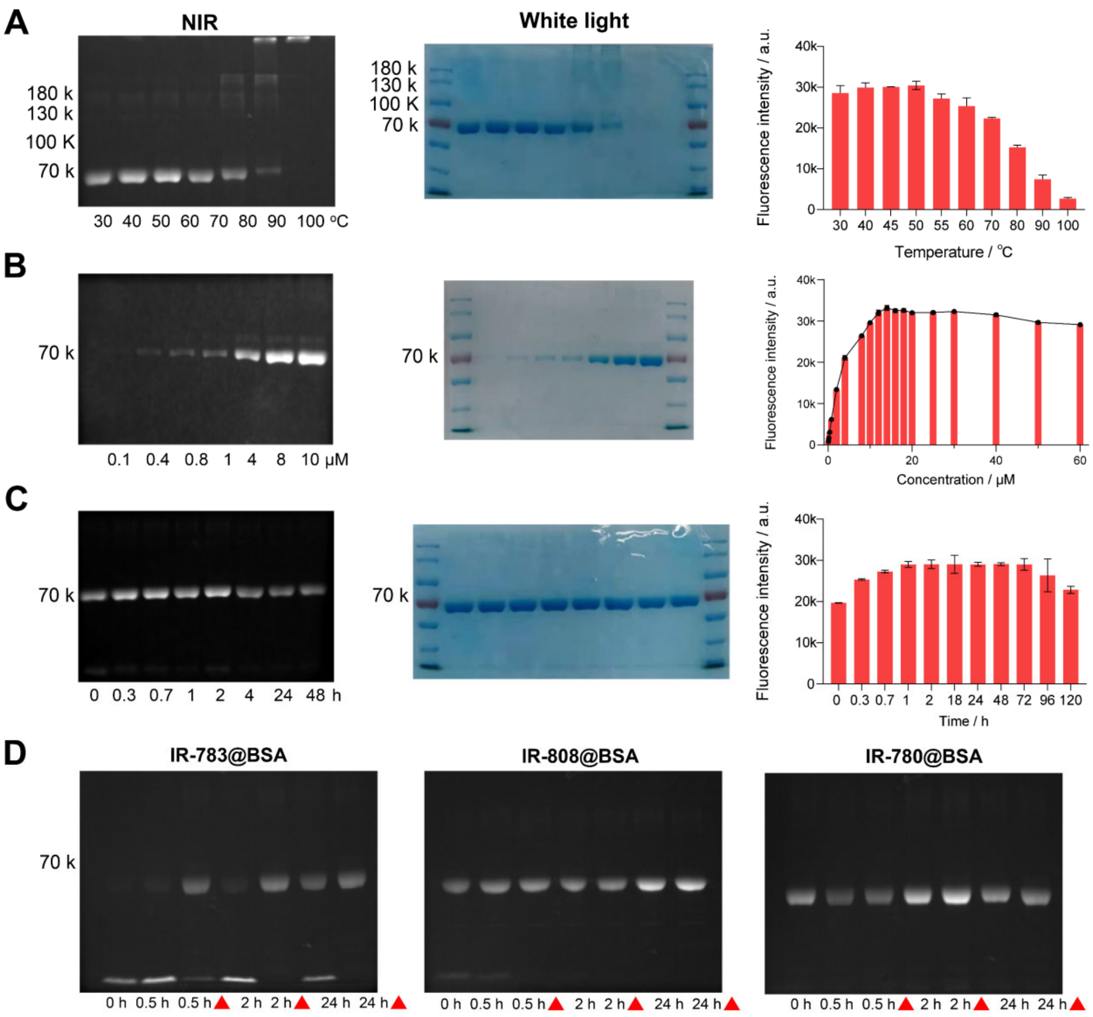

Figure S1. Reaction optimization for IR-780@BSA fluorophore. Electrophoresis gel analysis and brightness of IR-780@BSA for optimizing (A) reaction temperature (10 μM, 2 h, 1:1), (B) concentration (50 °C, 2 h, 1:1), and (C) time (50 °C, 10 μM, 1:1). (D) The brightness of IR-783@BSA, IR-808@BSA, and IR-780@BSA with or without heating at increasing time points. All NIR-II images were captured with 900+1000 nm long-pass filters under 808 nm laser irradiation (70-75 mW/cm^2).

Data note:

1. The incubation temperature was limited in 30-60 °C, as overheating would destroy the spatial structure of protein and form albumin aggregation.

2. There was an approximately positive linear relationship between brightness and concentration under the concentration of 10 μM.

3. 2 h reaction time was sufficient to form stable dye@protein fluorophores.

4. The basic condition of forming covalent bonds was 2 h heating for IR-783, 0.5 h heating or instead 2 h mixing for IR-808, while no heating was required and a short time mixing was sufficient for IR-780.

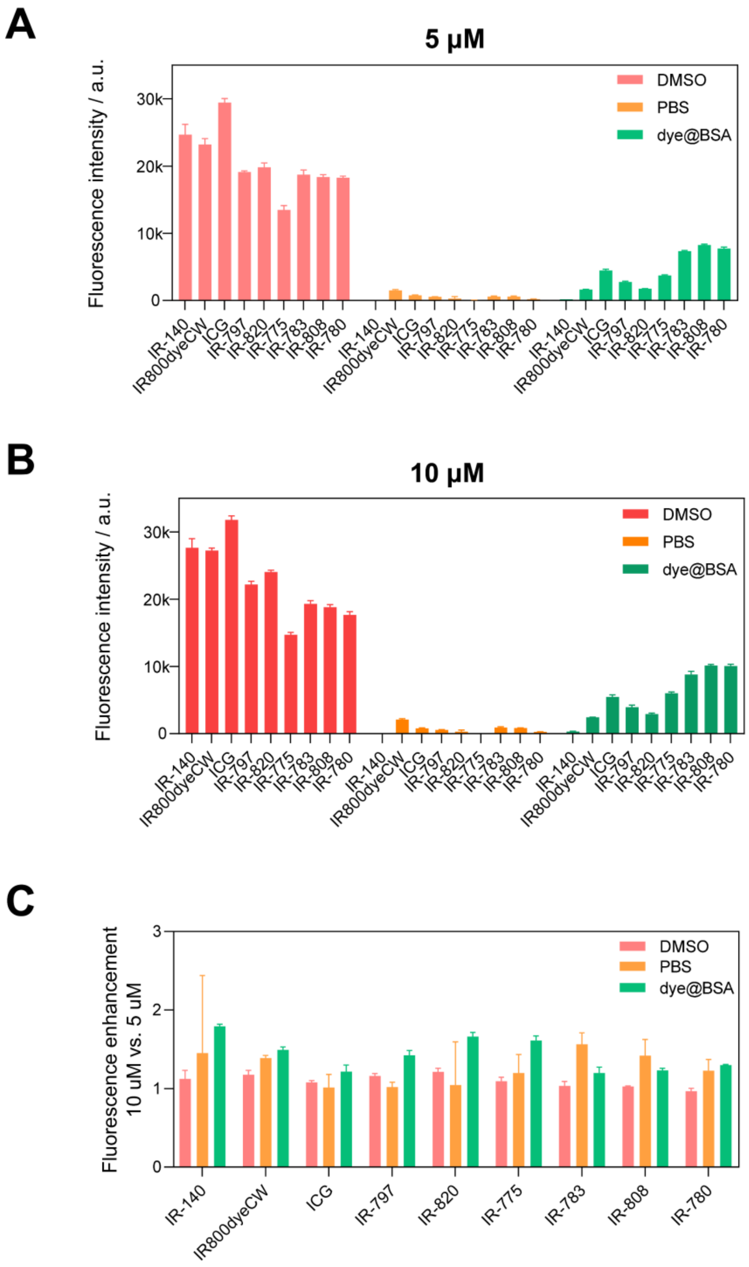

Figure S2. The brightness of free dye in DMSO, PBS, and dye@BSA fluorophores with concentrations (A) 5 and (B) 10 μM. (C) Fluorescence enhancement of dyes in DMSO, PBS, and dye@BSA (10 μM versus 5 μM).

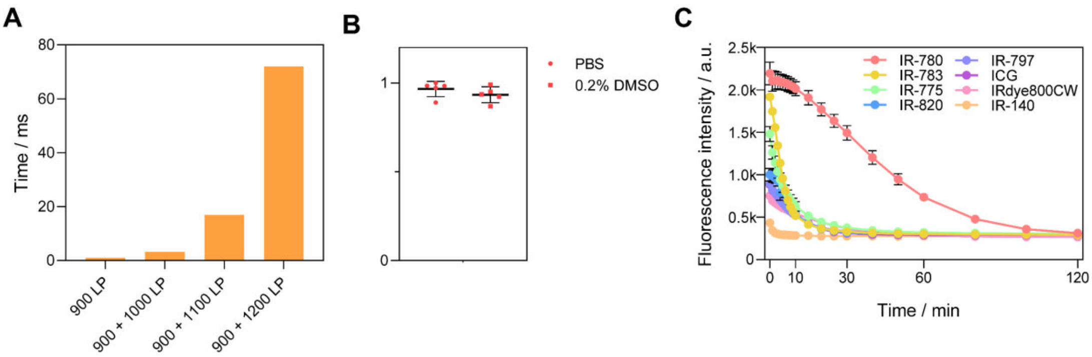

Figure S3. (A) Increasing exposure time for IR-780@BSA fluorophore could produce comparative brightness under the > 900, > 1000, > 1100, > 1200 nm sub-NIR-II windows. (B) The brightness of IR-780@BSA before and after removing 0.2% DMSO by ultrafiltration (30 kDa). (C) Photostability of dye@BSA fluorophores in the NIR-I window at a 1:1 reaction ratio under continuous laser irradiation.

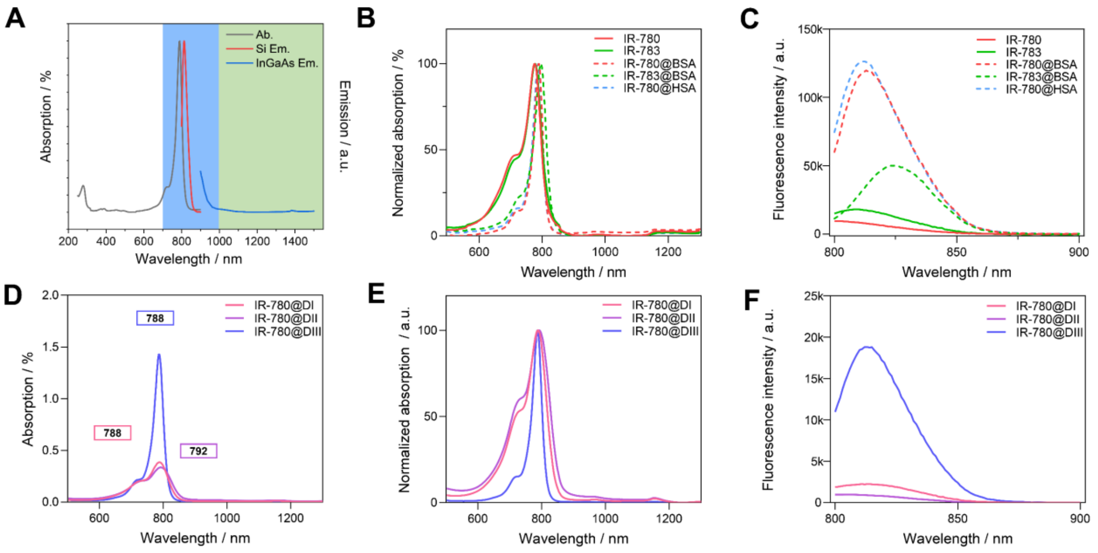

Figure S4. Absorption and emission spectra of free dyes and dye@protein fluorophores. (A) Absorption and NIR-I/NIR-II emission of 10 μM IR-780@BSA. Ab., absorption spectra; Si Em., emission spectra recorded on silicon camera; InGaAs Em., emission spectra recorded on InGaAs camera. (B) The normalized absorption spectra and (C) NIR-I emission spectra (790 nm excitation) of free dyes and dye@albumin fluorophores. (D) Absorption spectra, (E) the normalized absorption spectra, and (F) NIR-I emission spectra (790 nm excitation) of dye@albumin domain fluorophores.

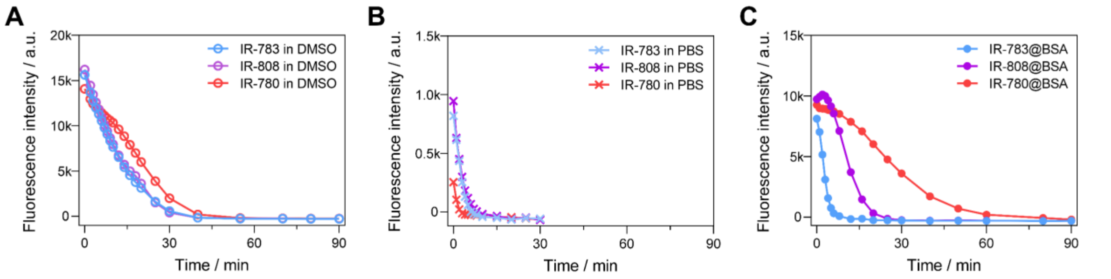

Figure S5. Photostability of IR-783, IR-808, and IR-780 in two buffers (DMSO, PBS) and their corresponding BSA fluorophores with the same dye concentration (10 µM) under continuous laser irradiation (808 nm, 70-75 mW/cm^2).

Data note:

IR-780@BSA fluorophore obtained much better photostability compared with IR-783@BSA and IR-808@BSA fluorophore, even the half-time period of IR-780@BSA was better than free dye in DMSO. The photostability of IR-780 in PBS was worse than that of IR-783 and IR-808, presumably due to the hydrophobicity feature of IR-780 causing the precipitation during the process of laser irradiation.

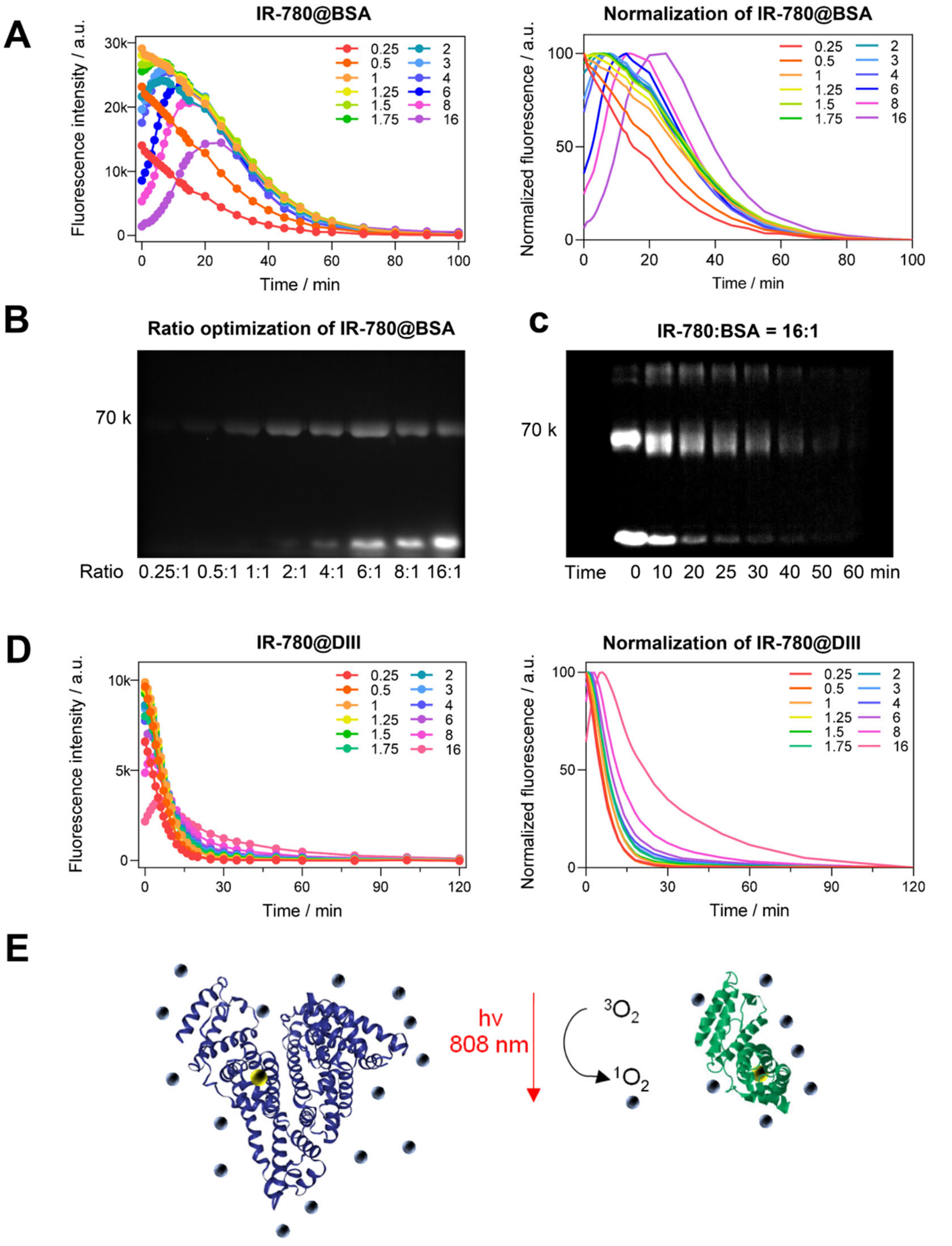

Figure S6. The photostability of IR-780@BSA and IR-780@DIII fluorophore. (A) Photostability and normalized fluorescence brightness of IR-780@BSA (10 μM) as a function of irradiation time (808 nm, 70-75 mW/cm^2). (B) Electrophoresis analysis of the fluorophores with IR-783 to BSA ratio from 0.25:1 to 16:1 (50 ^{O}C, 10 μM, 2 h). (C) Electrophoresis analysis of IR-780@BSA fluorophores (4 μM, IR-780:BSA=16:1) with increasing irradiation time under the continuous laser irradiation (808 nm, 70-75 mW/cm^2). (D) Photostability and normalized fluorescence brightness of IR-780@DIII as a function of irradiation time. (E) The simplified schematic diagram for the process of irradiating dye@BSA and dye@DIII: 808-nm laser irradiation could damage cyanine dyes through the generation of singlet oxygen, while the whole albumin provided better protection than domain III.

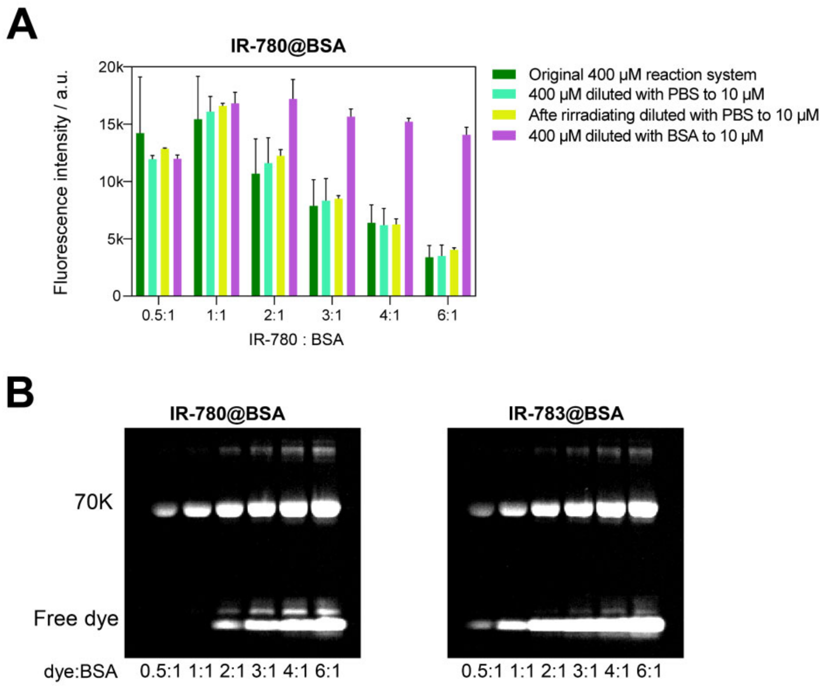

Figure S7. (A) The brightness of four groups of IR-780@BSA fluorophores: original 400 μM reaction system (400 μM), 400 μM reaction system diluted into PBS (10 μM), 400 μM reaction system irradiated for 2 h and then diluted into PBS (10 μM), and 400 μM reaction system diluted into BSA (10 μM). (B) The electrophoresis analysis of IR-780@BSA and IR-783@BSA with reaction ratios from 0.5:1 to 6:1 (diluted with PBS from 400 to 10 μM). All fluorescence intensity data and NIR-II images were performed with 900+1000 nm long-pass filters under 808 nm laser irradiation (60 mW/cm^2).

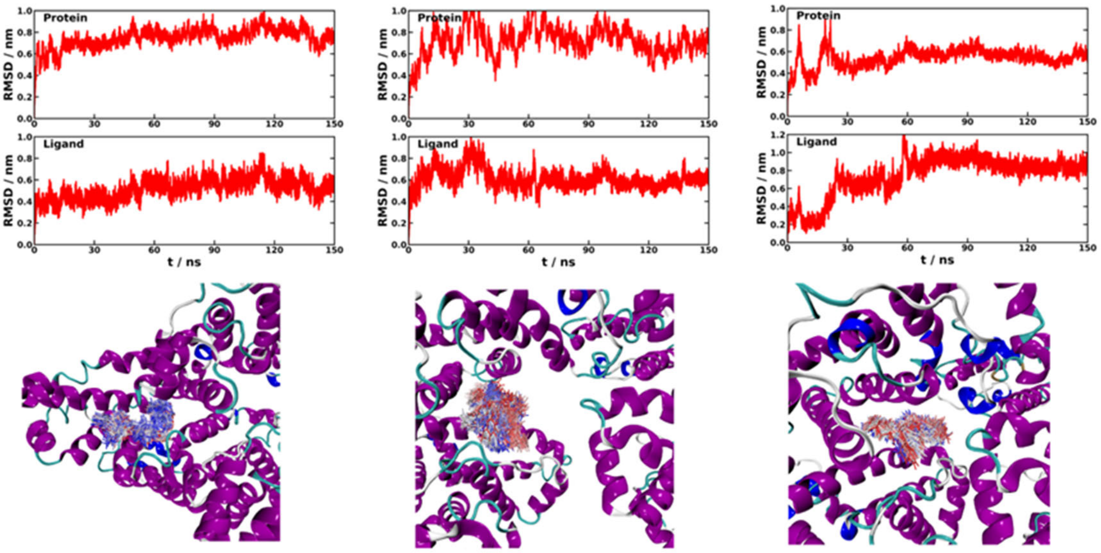

Figure S8. Root-mean-square deviations (RMSD) of protein and ligand in the MD simulation of 150 ns (top) and the overlap of 10000 snapshots taken from the equilibrium MD trajectories. From left to right: IR-780@BSA, IR-808@BSA, IR-783@BSA.

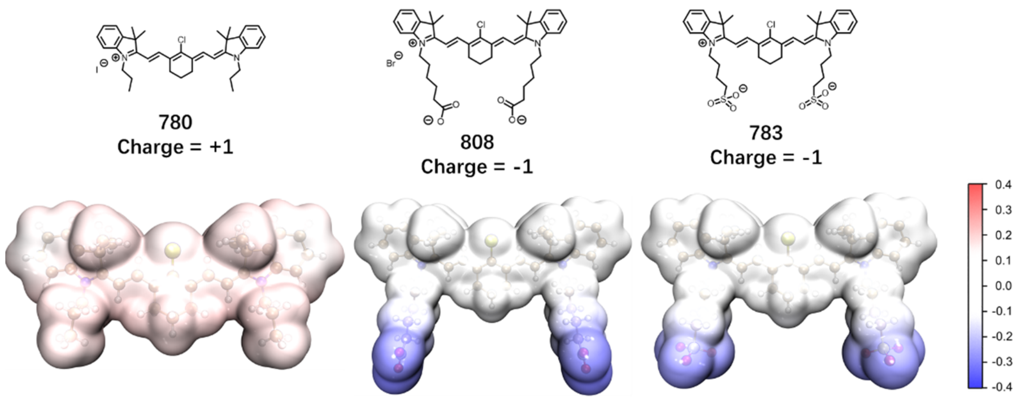

Figure S9. The positive charge distribution simulation of IR-780, IR-808, and IR-783.

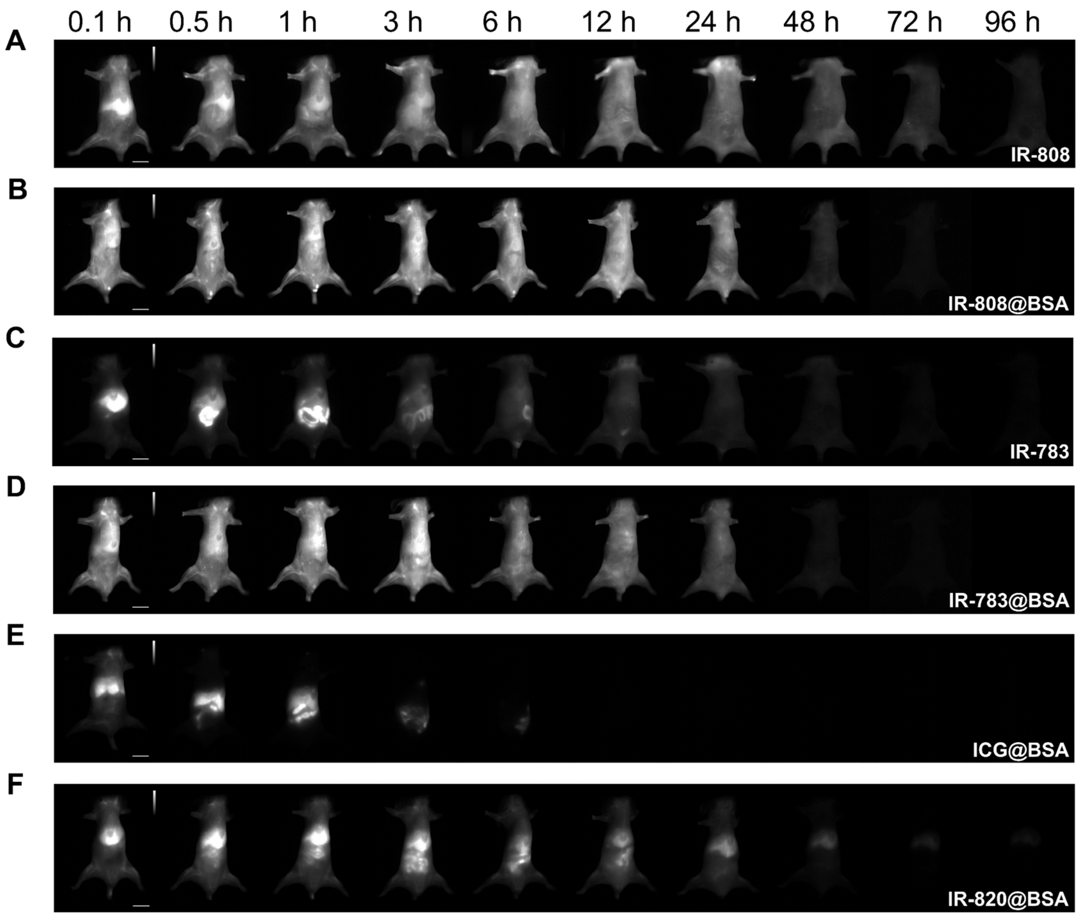

Figure S10. The metabolism behavior of typical free dyes and dye@BSA fluorophores. Whole-body imaging with (A) IR-808, (B) IR-808@BSA, (C) IR-783, (D) IR-783@BSA, (E) ICG@BSA, and (F) IR-820@BSA (150 μM, 200 μL) at increasing time points after tail vein administration of these probes. The scale bar presents 1 cm. All NIR-II images were captured with 1000+1100 nm long-pass filters under 808 nm laser irradiation (60 mW/cm^2). Exposure time was 20 ms. The fluorescence intensity scale bar of A-F ranges from 0−20000, 0−25000, 0−20000, 0−15000, 0−40000, and 0−40000, respectively.

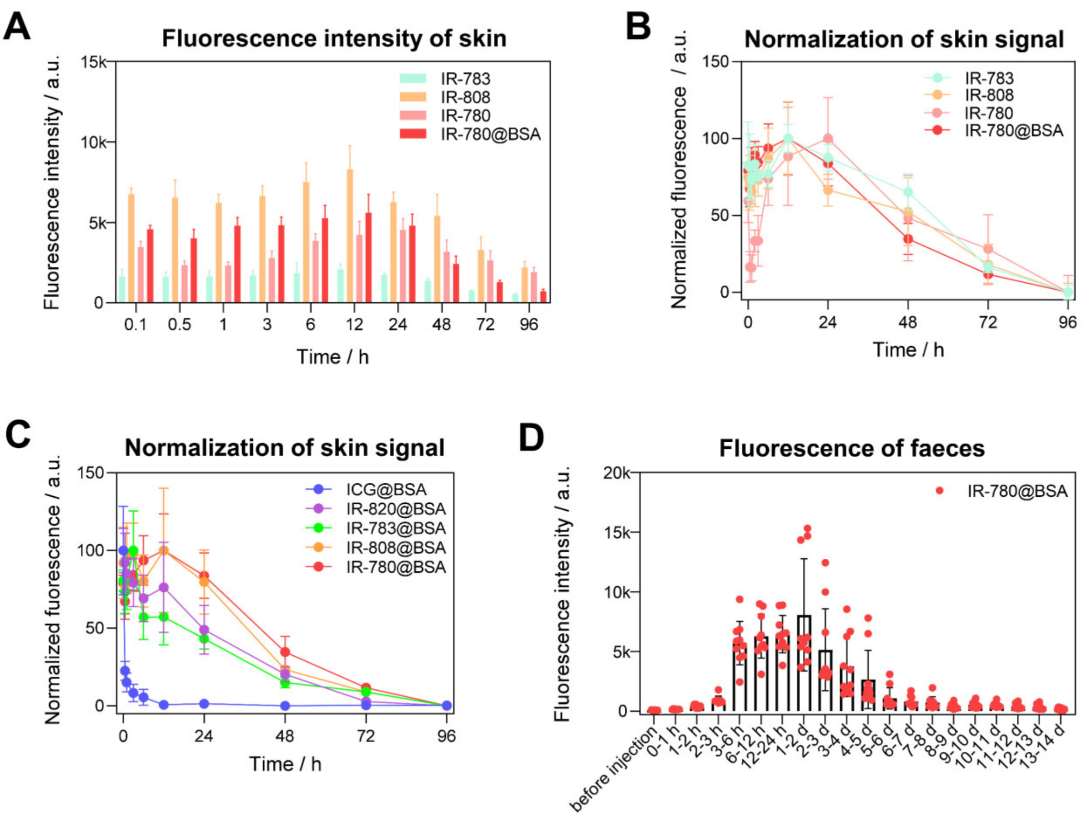

Figure S11. Statistical analysis of skin (or skin + muscle) fluorescence intensity of free dye and dye@BSA fluorophores injected mice with increasing time points. (A) Skin signal intensity of free dyes and IR-780@BSA injected mice. (B) Representative normalized skin signal intensity of free dyes and IR-780@BSA fluorophore. (C) Representative normalized skin signal of several typical dye@BSA fluorophores. (D) The fluorescence intensity of faeces excreted by IR-780@BSA injected mice.

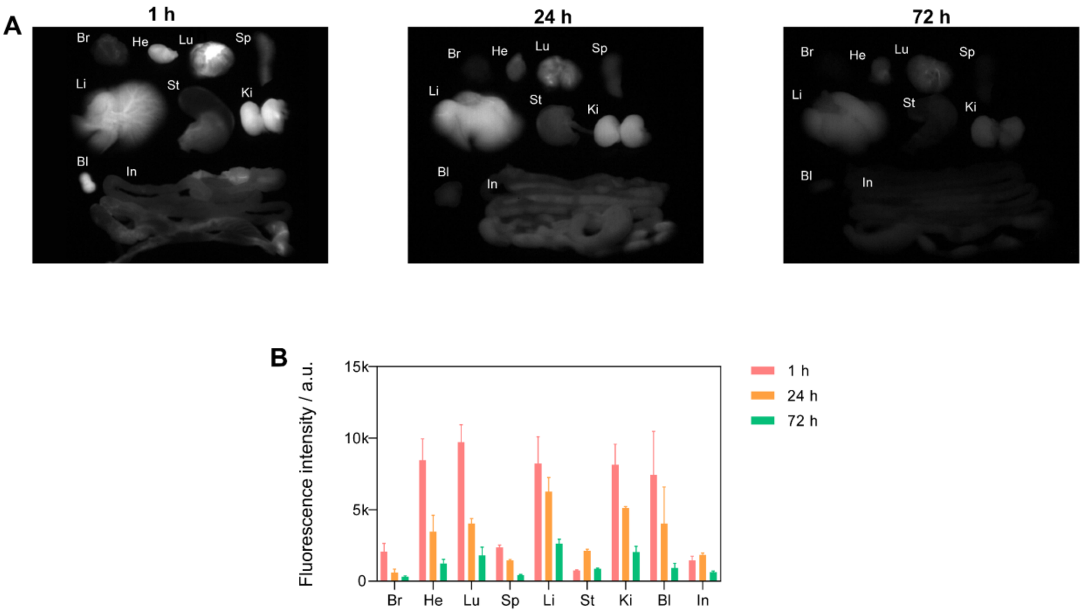

Figure S12. (A) Ex vivo fluorescence images of organs dissected from mice after injecting IR-780@BSA at 1, 24, and 72 h time points. (B) The fluorescence intensity was plotted based on the imaging data from A. The fluorescence intensity scale bar of A ranges from 0−10000. Br, He, Lu, Sp, Li, St, Ki, Bl, and In are abbreviation for brain, heart, lung, spleen, liver, stomach, kidney, blood, and intestines, respectively.

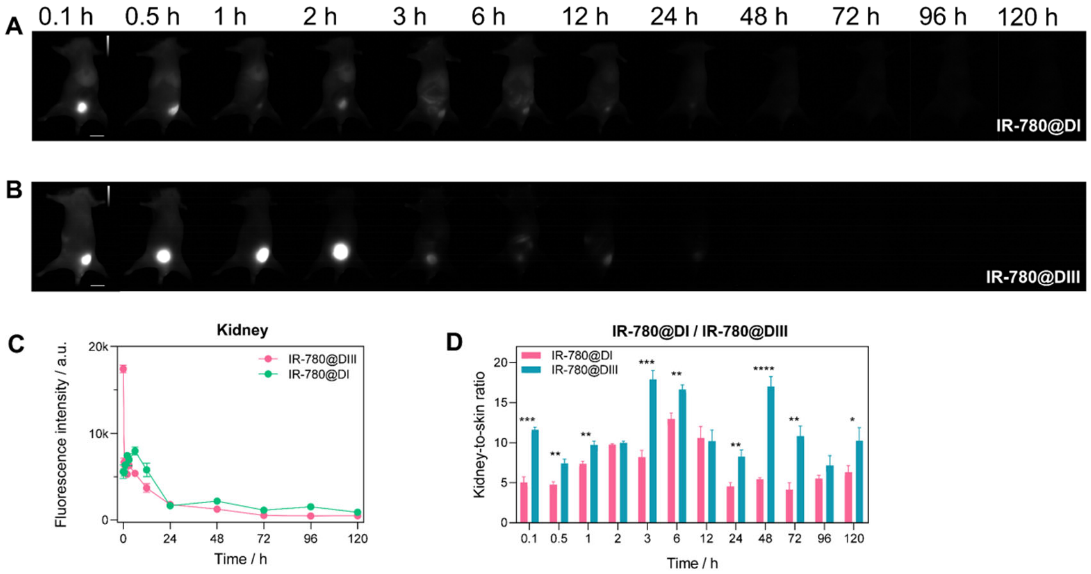

Figure S13. Comparison of the whole-body imaging of IR-780@DI and IR-780@DIII. (A-B) Selected time points of NIR-II imaging of mice in the supine position after intravenous administration of IR-780@DI and IR-780@DIII (150 µM, 200 µL), respectively. The imaging condition was the same with Figure 6C, D. Scale bar presents 1 cm. (C) Fluorescence intensity of kidney as a function of injection time for IR-780@DI and IR-780@DIII. (D) Statistical analysis for the kidney-to-skin ratio of IR-780@DI and IR-780@DIII. The fluorescence intensity scale bar of A and B ranges from 0-10000.

Table S1. The tested solubility of each dye in DMSO, PBS, and dye@BSA fluorophores.

Dye	DMSO	PBS	dye@BSA
IR-140	5000 µM	indissolvable	indissolvable
IRdye800CW	5000 µM	600 µM	600 µM
ICG	5000 µM	600 µM	600 µM
IR-797	5000 Mm	150 µM	500 µM
IR-820	5000 µM	600 µM	600 µM
IR-775	5000 µM	275 µM	500 µM
IR-783	5000 µM	600 µM	600 µM
IR-808	5000 µM	600 µM	600 µM
IR-780	5000 µM	275 µM	500 µM

Data note:

1. Please noted that the tested concentrations are not their maximum solubility. All the tested solubility concentrations were much higher than we actually used in this work.
2. The tested solubility concentration of dyes in PBS and dye@BSA was chosen as 600 µM because the generally used concentration of BSA is 600 µM (~40 mg/mL).

Table S2. Calculated MM/GBSA Protein-Ligand Binding Free Energy (kcal/mol) for the three protein-ligand fluorophores, i.e., IR-780@BSA, IR-808@BSA, and IR-783@BSA.

Samples: 10000 frames in 1 ns with an sample frequency of 100 fs

Fluorophore	ΔG_{gas}	ΔG_{sol}	ΔG_{total}	$\Delta\Delta G_{total}$
780@BSA	-249.46	201.90	-47.56	-11.42
808@BSA	5.85	-47.98	-42.13	-5.99
783@BSA	40.36	-76.50	-36.14	0

Data note:

1. ΔGgas is defined as the difference of gas-phase energy between that of the protein−ligand fluorophore and those of the separate protein and ligand. The AMBER99SB-ILDN for protein and GAFF force field with RESP2 charge for ligand are used.
2. ΔGsol is the difference of solvation free energy between that of the protein−ligand fluorophore and those of the separate protein and ligand systems. The solvation free energy is computed with the GBSA method. In the GBSA calculation, the interior and exterior dielectric constants are set to 1 and 80, respectively.
3. ΔGtotal = ΔGgas + ΔGsol is the protein-ligand binding free energy without the entropic contribution (-TΔS). Since a reasonable entropic contribution is hard to get and the main contribution of binding free energy comes from enthalpy, the entropic contribution is not included in the binding free energy (assuming the entropic contributions of these three fluorophores are similar).
4. All the calculations are performed with the gmx_MMPBSA tool based on AMBER's MMPBSA.py.

References

1. Tian R, Zeng Q, Zhu S, Lau J, Chandra S, Ertsey R, et al. Albumin-chaperoned cyanine dye yields superbright NIR-II fluorophore with enhanced pharmacokinetics. Sci Adv. 2019; 5: eaaw0672.
2. Frisch MJ, Trucks GW, Schlegel HB, Scuseria GE, Robb MA, Cheeseman JR, et al. Gaussian 16 Rev. B.01. Wallingford, CT; 2016.
3. Tawada Y, Tsuneda T, Yanagisawa S, Yanai T, Hirao K. A long-range-corrected time-dependent density functional theory. J Chem Phys. 2004; 120: 8425-33.
4. Krishnan R, Binkley JS, Seeger R, Pople JA. Self-consistent molecular orbital methods. XX. A basis set for correlated wave functions. J Chem Phys. 1980; 72: 650-4.
5. Clark T, Chandrasekhar J, Spitznagel GW, Schleyer PVR. Efficient diffuse function-augmented basis sets for anion calculations. III. The 3-21+G basis set for first-row elements, Li–F. J Comput Chem. 1983; 4: 294-301.
6. Spitznagel GW, Clark T, von Ragué Schleyer P, Hehre WJ. An evaluation of the performance of diffuse function-augmented basis sets for second row elements, Na-Cl. J Comput Chem. 1987; 8: 1109-16.
7. Sun H, Autschbach J. Influence of the Delocalization Error and Applicability of Optimal Functional Tuning in Density Functional Calculations of Nonlinear Optical Properties of Organic Donor–Acceptor Chromophores. ChemPhysChem. 2013; 14: 2450-61.
8. Sun H, Zhong C, Brédas J-L. Reliable Prediction with Tuned Range-Separated Functionals of the Singlet–Triplet Gap in Organic Emitters for Thermally Activated Delayed Fluorescence. J Chem Theory Comput. 2015; 11: 3851-8.
9. Tomasi J, Mennucci B, Cammi R. Quantum Mechanical Continuum Solvation Models. Chem Rev. 2005; 105: 2999-3094.
10. Bayly CI, Cieplak P, Cornell W, Kollman PA. A well-behaved electrostatic potential based method using charge restraints for deriving atomic charges: the RESP model. J Phys Chem. 1993; 97: 10269-80.
11. Schauperl M, Nerenberg PS, Jang H, Wang L-P, Bayly CI, Mobley DL, et al. Non-bonded force field model with advanced restrained electrostatic potential charges (RESP2). Commun Chem. 2020; 3: 44.
12. Lu T, Chen F. Multiwfn: A multifunctional wavefunction analyzer. J Comput Chem. 2012; 33: 580-92.
13. Wang J, Wolf RM, Caldwell JW, Kollman PA, Case DA. Development and testing of a general amber force field. J Comput Chem. 2004; 25: 1157-74.
14. Sousa da Silva AW, Vranken WF. ACPYPE - AnteChamber PYthon Parser interfacE. BMC Res Notes. 2012; 5: 367.
15. Burley SK, Bhikadiya C, Bi C, Bittrich S, Chen L, Crichlow GV, et al. RCSB Protein Data Bank: powerful new tools for exploring 3D structures of biological macromolecules for basic and applied research and education in fundamental biology, biomedicine, biotechnology, bioengineering and energy sciences. Nucleic Acids Res. 2021; 49: D437-D51.
16. Bujacz A. Structures of bovine, equine and leporine serum albumin. Acta Crystallogr D. 2012; 68: 1278-89.
17. Lindorff-Larsen K, Piana S, Palmo K, Maragakis P, Klepeis JL, Dror RO, et al. Improved side-chain torsion potentials for the Amber ff99SB protein force field. Proteins: Struct Funct Bioinformatics. 2010; 78: 1950-8.
18. Trott O, Olson AJ. AutoDock Vina: Improving the speed and accuracy of docking with a new

scoring function, efficient optimization, and multithreading. J Comput Chem. 2010; 31: 455-61.
19. Jorgensen WL, Chandrasekhar J, Madura JD, Impey RW, Klein ML. Comparison of simple potential functions for simulating liquid water. J Chem Phys. 1983; 79: 926-35.
20. Hess B, Bekker H, Berendsen HJC, Fraaije JGEM. LINCS: A linear constraint solver for molecular simulations. J Comput Chem. 1997; 18: 1463-72.
21. Essmann U, Perera L, Berkowitz ML, Darden T, Lee H, Pedersen LG. A smooth particle mesh Ewald method. J Chem Phys. 1995; 103: 8577-93.
22. Bussi G, Donadio D, Parrinello M. Canonical sampling through velocity rescaling. J Chem Phys. 2007; 126: 014101.
23. Berendsen HJC, Postma JPM, van Gunsteren WF, DiNola A, Haak JR. Molecular dynamics with coupling to an external bath. J Chem Phys. 1984; 81: 3684-90.
24. Parrinello M, Rahman A. Polymorphic transitions in single crystals: A new molecular dynamics method. J Appl Phys. 1981; 52: 7182-90.
25. Abraham MJ, Murtola T, Schulz R, Páll S, Smith JC, Hess B, et al. GROMACS: High performance molecular simulations through multi-level parallelism from laptops to supercomputers. SoftwareX. 2015; 1-2: 19-25.
26. Wang E, Sun H, Wang J, Wang Z, Liu H, Zhang JZH, et al. End-Point Binding Free Energy Calculation with MM/PBSA and MM/GBSA: Strategies and Applications in Drug Design. Chem Rev. 2019; 119: 9478-508.
27. Valdes-Tresanco MS, Valdes-Tresanco ME, Valiente PA, Moreno E. gmx_MMPBSA: A New Tool to Perform End-State Free Energy Calculations with GROMACS. J Chem Theory Comput. 2021; 17: 6281-91.
28. Miller BR, McGee TD, Swails JM, Homeyer N, Gohlke H, Roitberg AE. MMPBSA.py: An Efficient Program for End-State Free Energy Calculations. J Chem Theory Comput. 2012; 8: 3314-21.
